# Cxcr3 constrains pancreatic cancer dissemination through instructing T cell fate

**DOI:** 10.1007/s00262-022-03338-7

**Published:** 2022-12-06

**Authors:** Adam L. Burrack, Ellen J. Spartz, Meagan R. Rollins, Ebony A. Miller, Maria Firulyova, Eduardo Cruz, Michael F. Goldberg, Iris X. Wang, Hezkiel Nanda, Steven Shen, Konstantin Zaitsev, Ingunn M. Stromnes

**Affiliations:** 1 Department of Microbiology and Immunology, University of Minnesota Medical School, 2101 6th St SE, 2-186 WMBB, Minneapolis, MN 55414, USA; 2 Center for Immunology, University of Minnesota Medical School, Minneapolis, MN 55415, USA; 3 Computer Technologies Laboratory, ITMO University, Saint Petersburg, Russia; 4 Institute for Health Informatics, University of Minnesota Medical School, Minneapolis, MN 55414, USA; 5 Clinical Translational Science Institute, University of Minnesota, Minneapolis, MN, USA; 6 Masonic Cancer Center, Minneapolis, USA; 7 Center for Genome Engineering, University of Minnesota Medical School, Minneapolis, MN 55414, USA

**Keywords:** Pancreatic cancer, T cells, Cxcr3, Metastasis, PDA, PD-L1

## Abstract

Pancreatic ductal adenocarcinoma (PDA) is a lethal and metastatic malignancy resistant to therapy. Elucidating how pancreatic tumor-specific T cells differentiate and are maintained in vivo could inform novel therapeutic avenues to promote T cell antitumor activity. Here, we show that the spleen is a critical site harboring tumor-specific CD8 T cells that functionally segregate based on differential Cxcr3 and Klrg1 expression. Cxcr3+ Klrg1-T cells express the memory stem cell marker Tcf1, whereas Cxcr3-Klrg1 + T cells express GzmB consistent with terminal differentiation. We identify a Cxcr3+ Klrg1+ intermediate T cell subpopulation in the spleen that is highly enriched for tumor specificity. However, tumor-specific T cells infiltrating primary tumors progressively downregulate both Cxcr3 and Klrg1 while upregulating exhaustion markers PD-1 and Lag-3. We show that antigen-specific T cell infiltration into PDA is Cxcr3 independent. Further, Cxcr3-deficiency results in enhanced antigen-specific T cell IFNγ production in primary tumors, suggesting that Cxcr3 promotes loss of effector function. Ultimately, however, Cxcr3 was critical for mitigating cancer cell dissemination following immunotherapy with CD40 agonist + anti-PD-L1 or T cell receptor engineered T cell therapy targeting mesothelin. In the absence of Cxcr3, splenic Klrg1 + GzmB + antitumor T cells wain while pancreatic cancer disseminates suggesting a role for these cells in eliminating circulating metastatic tumor cells. Intratumoral myeloid cells are poised to produce Cxcl10, whereas splenic DC subsets produce Cxcl9 following immunotherapy supporting differential roles for these chemokines on T cell differentiation. Together, our study supports that Cxcr3 mitigates tumor cell dissemination by impacting peripheral T cell fate rather than intratumoral T cell trafficking.

## Introduction

The incidence and mortality of pancreatic ductal adenocarcinoma (PDA) is on the rise [1] and predicted to become the 2nd leading cause of cancer-related deaths by 2030 [2]. While immunotherapies elicit clinical responses in many malignancies [3, 4], PDA is often resistant [5]. The robust fibroinflammatory tumor microenvironment (TME) characteristic of PDA is believed to limit T cell access, functionality and immunotherapy response [6]. However, our studies in faithful PDA animal models support that antigen-specific T cells preferentially accumulate in primary tumors and metastasis [7, 8]. Thus, understanding how T cells can access malignant sites and the factors that contribute to maintenance of functional antitumor T cells will inform effective immunotherapy design.

Chemokines are small, secreted proteins that instruct cellular migration through venules into and out of tissues. Cxcr3 is a chemokine receptor expressed by effector and memory T cells and binds interferon-inducible chemokines including Cxcl9 and Cxcl10 [9, 10]. Cxcr3 promotes T cell migration [11, 12] and PD-1 blockade efficacy [13, 14] in subcutaneous implantable tumor animal models. Cxcr3 and its ligands are associated with a T cell inflamed signature in many cancers [12] and plasma levels of Cxcl9 and Cxcl10 are associated with prolonged survival in PDA patients treated with chemotherapy [15]. Cxcr3 promotes effector T cell (T_EFF_) fate at the expense of central memory T cells (T_CM_) following a primary immune response to infection [16–19]. After viral infection, Cxcr3 guides CD8 T cells to the lymph node periphery promoting effector T cell differentiation [19]. During chronic *T. gondii* infection in mice, Cxcr3 regulates the differentiation of antigen-specific Cxcr3 + Klrg1− memory T cells into Cxcr3+ Klrg1+ intermediate effector/memory T cells in the spleen [20]. However, the role of Cxcr3 on a tumor-specific T cell response in PDA is unknown.

In contrast to the potential antitumor roles for Cxcr3, intratumoral Cxcr3 ligands are associated with a worse PDA patient prognosis [21]. Cxcr3 is expressed on a subset of Foxp3 + regulatory T cells (Treg) [22] and Cxcl10 expression by cancer-associated fibroblasts can recruit suppressive Treg [23]. Pancreatic tumor cells can also express Cxcr3, and Cxcr3:Cxcl10 interactions facilitate tumor cell migration to sensory neurons and contribute to cancer-related pain [24]. Thus, Cxcr3 has the potential to promote or mitigate PDA pathogenesis.

We previously showed that conventional type 1 dendritic cells (cDC1s) are critical for maintaining both spleen-residing and intratumoral tumor-specific T cells in PDA [25]. As cDC1s have been previously shown to produce Cxcr3 ligands in subcutaneous tumor models [13], we posited that the Cxcr3 pathway may play a role in tumor-specific T cell fate during pancreatic cancer growth and immunotherapy. We identify that both endogenous and adoptively transferred TCR engineered antigen-specific T cell infiltration into PDA is Cxcr3 independent. However, our study supports that Cxcr3 is critical for immunotherapy durability through maintaining peripheral GzmB + Klrg1 + effector T cells deemed critical for mitigating cancer cell dissemination. In primary tumors, Cxcr3 instead promotes T cell exhaustion. Thus, our study reveals a link between peripheral effector T cell differentiation and exhaustion in the tumor bed and tissue-specific roles for Cxcr3 on T cell differentiation and antitumor immunity.

## Results

### Cxcr3 and Klrg1 delineate endogenous tumor-specific T cell subpopulations during PDA progression

**Fig. 1:**
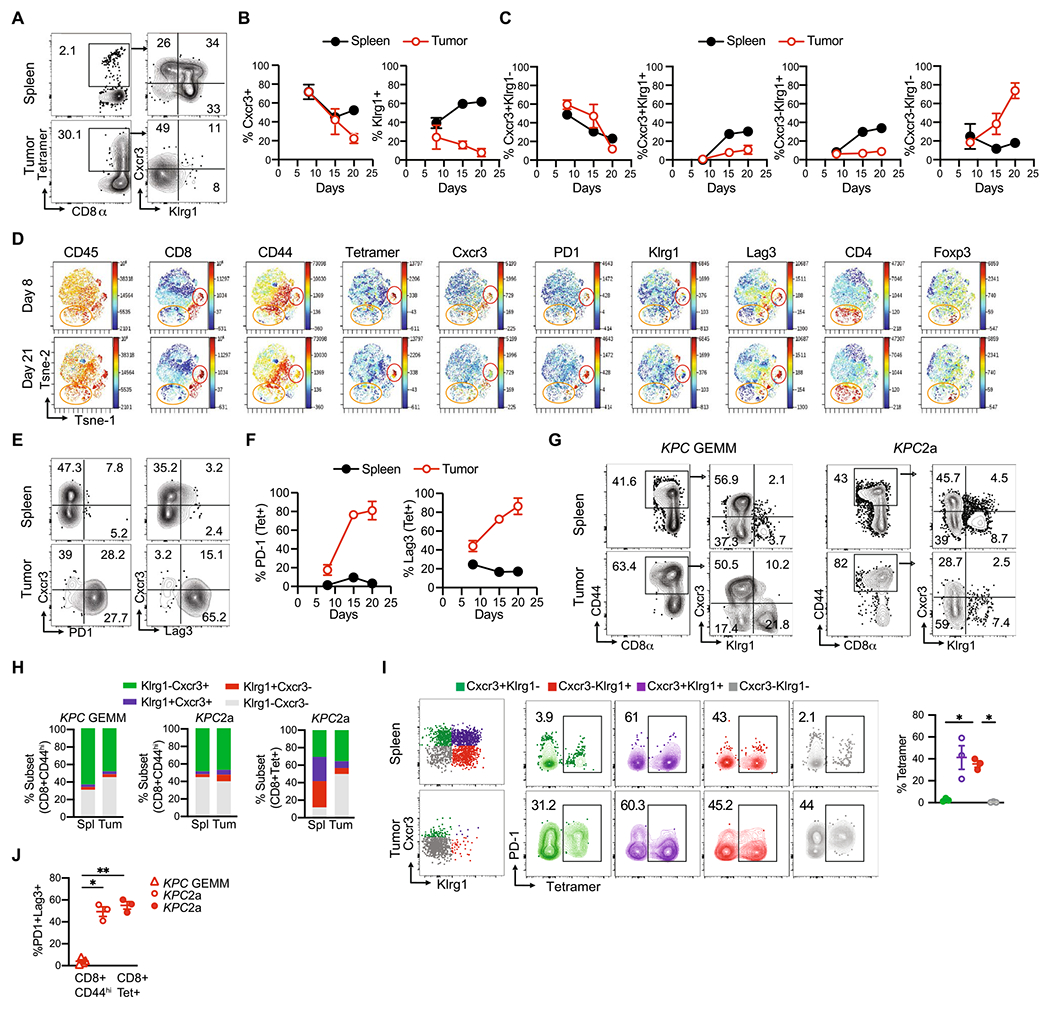
Cxcr3 and Klrg1 delineate endogenous tumor-specific T cell subpopulations during PDA progression. **A** Representative tetramer staining and gating strategy (left) and Cxcr3 and Klrg1 expression (right, gated on CD8 + tetramer + T cells) on day 21 post orthotopic *KPC*2a tumor implantation. **B** Frequency of Cxcr3+ or Klrg1 + tetramer + T cells gated on total CD8 + tetramer + T cells following *KPC*2a orthotopic tumor implantation. Data are mean ± S.E.M. *n* = 4–5 mice per timepoint. **C** Frequency of the indicated subsets gated on CD8 + tetramer + T cells. Data are mean ± S.E.M. *n* = 4–5 mice per timepoint. **D** ViSNE analysis of *n* = 3–4 concatenated samples per timepoint from *KPC*2a-bearing mice gated on live CD45 + cells. Red circle, CD8 + tetramer + cells. Orange circle, CD4 + T cells. **E** Representative plots of Cxcr3, PD1 and Lag3 on CD8 + tetramer + T cells on day 21 posttumor. **F** Frequency of tetramer + T cells that express PD-1 or Lag3 in *KPC*2a tumor-bearing mice. Data are mean ± S.E.M. *n* = 4–5 mice per timepoint. **G** Representative gating strategy for analyzing Cxcr3 and Klrg1 proportions among CD44^hi^ CD8+ T cells in the *KPC* GEMM or the *KPC*2a orthotopic model. *n* = 3 mice per group. **H** Mean proportion of each subset among the indicated T cell population and model. *n* = 3 mice per group. **I** Gating strategy for assessing Cxcr3 and Klrg1 as markers to identify tetramer-binding T cells at 2 weeks post *KPC*2a orthotopic tumor implantation. Data are quantified (right) and are mean ± S.E.M. *n* = 3 mice per group. **J** Frequency of antigen-experienced (CD8 + CD44^high^) T cells or CD8 + tetramer + that co-express PD-1 and Lag-3 from tumors in the *KPC* GEMM or the *KPC*2a orthotopic model

Elucidating how pancreatic tumor-specific T cells differentiate and are maintained in vivo could inform novel therapeutic avenues to promote their antitumor activity. Indeed, the phenotypic traits and qualities of tumor-specific T cells in secondary lymphoid organs as well as intratumorally in PDA are largely unclear. To investigate tumor-specific T cell differentiation during PDA progression, we employed the orthotopic *KPC*2a model in which tumor cells express click-beetle red luciferase (CB) that serves for tumor imaging [26, 27] and as a model neoantigen [8, 25, 28]. Tumor (CB)-specific T cells can be tracked longitudinally in vivo with a fluorescent CB_101-109_:H-2D^b^ tetramer (Fig. 1A) [8, 25, 28]. *KPC* cells that express ovalbumin are rejected in syngeneic immunocompetent mice after orthotopic implantation [8] rendering the CB + tumor model a valuable alternative to study tumor-specific T cells. As T cell phenotype can identify similar differentiation states in diverse biological contexts, we assessed Cxcr3 and Klrg1 as landmark markers because functionally distinct pathogen-specific CD8 T cells can be distinguished based upon Cxcr3 and Klrg1 during chronic infection [20, 29]. Specifically, Cxcr3+ Klrg1− T cells exhibit sustained proliferative capacity and differentiate into Cxcr3+ Klrg1+ T cells, which give rise to terminally differentiated Cxcr3-Klrg1 + T cells during chronic infection [20, 29]. Therefore, we posited a similar differentiation pattern may be maintaining tumor-specific T cells. We identified that 60–80% of CB_101-109_:H-2D^b^ tetramer + T cells expressed Cxcr3 in both spleen and tumor on day 7 posttumor (Fig. 1B). While 50–60% of splenic tetramer + T cells were Cxcr3+ until day 21, intratumoral tetramer + T cells progressively downregulated Cxcr3 (Fig. 1A–B). Klrg1+ tetramer + T cell frequency progressively increased in spleen and decreased in tumor (Fig. 1B). Cxcr3+ Klrg1-tetramer + T cells frequency progressively decreased in spleen and tumor over time (Fig. 1C). Within spleens, this corresponded to an increase in putatively more differentiated Cxcr3+ Klrg1 + and Cxcr3-Klrg1 + subpopulations (Fig. 1C). In contrast, the progressive loss of Cxcr3+tetramer + T cells in tumor corresponded to an accumulation of Cxcr3-Klrg1-cells (Fig. 1C), a subset not previously identified in the chronic infection model [20, 29]. To identify the differentiation state of Cxcr3-Klrg1-tetramer + T cells in PDA, we performed ViSNE analysis on CD45 + cells. Intratumoral Cxcr3-Klrg1-tetramer + T cells exhibited elevated PD-1 and Lag-3 (Fig. 1D–F), markers of PDA-specific T cells defective in IFNγ and GzmB [8, 25, 28] and consistent with an acquisition of an exhausted T (T_EX_) cell state. ViSNE analysis showed Cxcr3 was mostly confined to T cells in murine PDA including a fraction of CD4 + Foxp3 + (Treg) and CD4 + Foxp3-T cells (Fig. 1D). In non-tumor-bearing mice, Cxcr3 was expressed by memory and effector T cells but not naïve T cells (Supplementary Fig. 1A–C), as expected [9]. Cxcr3 was also expressed by antigen experienced CD44 + CD4 + T cells (Supplementary Fig. 1D), maintained in spleen and progressively decreased on intratumoral Treg and CD4 + Foxp3− T cells (Supplementary Fig. 1E).

The data above suggest that Cxcr3 and Klrg1 may define distinct T cell differentiation states during invasive tumor growth. However, one limitation of the above approach was the highly aggressive orthotopic model of invasive PDA fails to recapitulate the progression of PDA through a preinvasive state nor the robust fibroinflammatory response characteristic of the human disease. Therefore, we next investigated if phenotypically similar subsets were present in the autochthonous *KPC* genetically engineered mouse model (GEMM) of PDA. Since there are no known immunogenic epitopes in the *KPC* GEMM, we could not distinguish tumor-specific from non-specific T cells. Therefore, we gated on total CD8 + CD44^hi^ antigen-experienced T cells in spleen and tumor from the *KPC* GEMM and the *KPC*2a model (Fig. 1G), with the understanding that this approach includes non-specific T cells. Despite this caveat, both Cxcr3+ Klrg1− and Cxcr3-Klrg1+ subsets were clearly present and at similar proportions in the two models (Fig. 1G–H). In contrast, in the spleen, the Cxcr3+ Klrg1+ intermediate population was quite rare (Fig. 1G–H), especially when comparing to the proportion of Cxcr3+ Klrg1+ tumor-specific (tetramer-binding) T cells (Fig. 1H). These data suggest that the Cxcr3 + Klrg1 + intermediate subpopulation in the spleen may be particularly enriched for tumor specificality. To test this hypothesis, we quantified the percentage of each Cxcr3/Klrg1 subset that bound tetramer in *KPC*2a tumor-bearing mice. Indeed, the Klrg1 + Cxcr3 + subset in the spleen was the most enriched for tetramer + T cells, with approximately 60% binding the CB_101-109_:H-2D^b^ tetramer (Fig. 1I). As expected, there were very few PD-1 + Lag3 + T cells infiltrating tumors in the *KPC* GEMM (Fig. 1J), consistent with a lack of tumor antigen specificity and a distinction from the *KPC*2a model (Fig. 1J).

We next evaluated *CXCR3* cellular distribution in human tissues. As expected, *CXCR3* and *KLRG1* were decreased in tumor vs. spleen (Supplementary Fig. 1F). Analysis of 6 merged human PDAs using a public scRNAseq dataset [30] showed that *CXCR3* was mostly confined to T cells (Supplementary Fig. 1G), similar to our results in mice. A minor *LILRA4* + population (cluster 21), which are likely plasmacytoid dendritic cells (DCs) [31], were also *CXCR3*+ in human PDA (Supplementary Fig. 1G). To identify T cell differentiation states in human PDA, we reclustered just *CD3E* + cells (Supplementary Fig. 1H). Cluster 0 contained *CD8A* + *CXCR3*+ *KLRG1*− T cells; Cluster 1 contained *CD4* + *FOXP3* + *TREG*; Cluster 2 contained *CD8A* + *CXCR3*+ *KLRG1*^*High*^ T cells; Cluster 3 contained *CD4* + *CCR7* + naïve or memory T cells; Cluster 4 contained *NCR1* + *NKG7* + NK cells; and Cluster 5, which was rare, contained C*D8A* + cells that were *CXCR3*^+^*KLRG1*^*Int*^. *PDCD1* and *LAG3* appeared enriched in CD8 T cell clusters 2 and 5 potentially reflective of T_EX_ (Supplementary Fig. 1I). Cluster 2 that was highest for *KLRG1* (Supplementary Fig. 1 J) was enriched for *GZMA* and *GZMK* suggesting enhanced cytotoxicity and an effector T cell differentiation state. Thus, the above markers may also be useful for delineating human T cell differentiation states in cancer.

### Cxcr3 is dispensable for intratumoral antigen-specific T cell accumulation

**Fig. 2:**
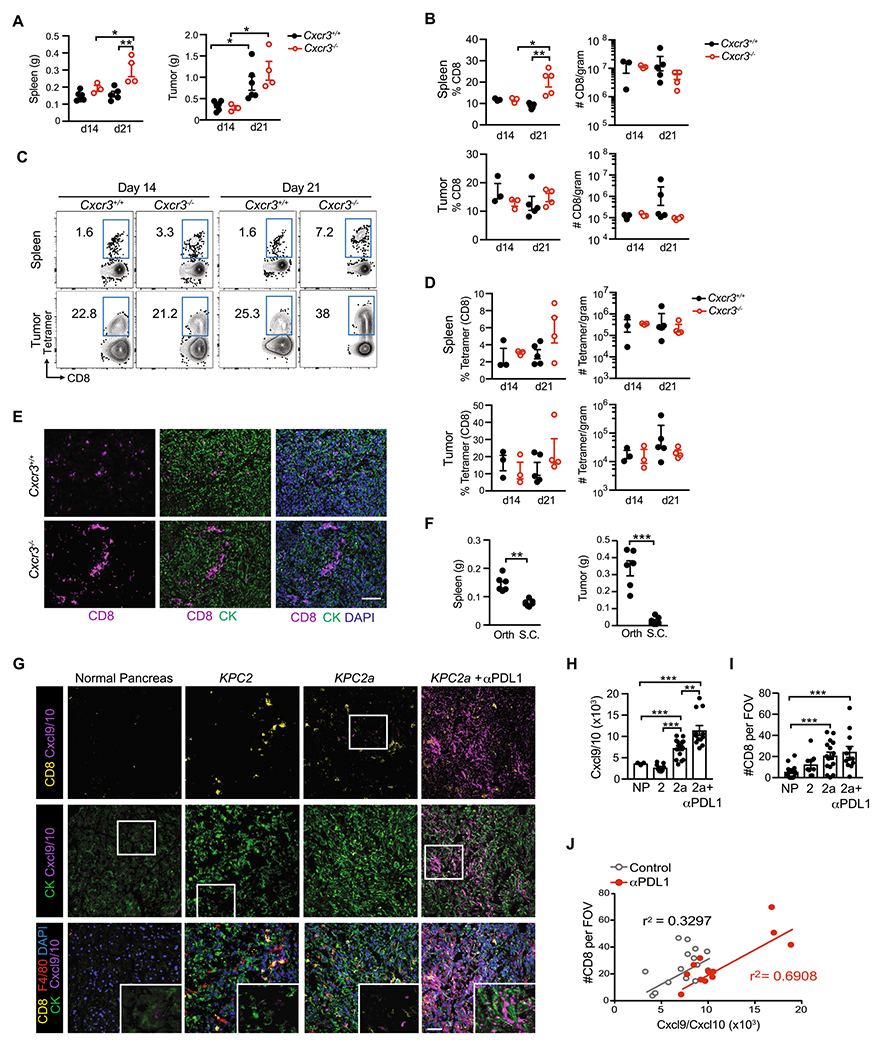
Cxcr3 is dispensable for intratumoral antigen-specific T cell accumulation. **A** Orthotopic *KPC*2a tumor and spleen weights on 14 or 21 days posttumor in *Cxcr3*^+/+^ and *Cxcr3*^−/−^ mice. Each dot is an independent animal. Data are mean ± S.E.M. *p < 0.05, **p < 0.005, ANOVA with a Tukey’s posttest. **B** Frequency and number of CD8 + T cells per gram spleen or tumor from *KPC*2a-bearing *Cxcr3*^+/+^ and *Cxcr3*^−/−^ mice on day 14 (d14) or day 21 (d21) posttumor. Each dot is an independent animal. Data are mean ± S.E.M. n = 3–4 mice per group. *p < 0.05, **p < 0.005, ANOVA with a Tukey’s posttest. **C** Representative H-2D^b^:CB_101-109_ tetramer staining gated on live CD8 + T cells infiltrating tumors from *KPC*2a-bearing *Cxcr3*^+/+^ and *Cxcr3*^−/−^ mice on day 14 or day 21 posttumor. **D** Frequency of tetramer + T cells of CD8 + T cells (left) and number of CD8 + tetramer + T cells per gram tissue. **E** Immunofluorescent detection of CD8 T cells infiltrating orthotopic tumors isolated from *Cxcr3*^+/+^ and *Cxcr3*^−/−^ mice on day 14. Scale bar, 50 μm. *n* = 3 mice per group. **F** Spleen and tumor weight from mice bearing orthotopic (Orth) or subcutaneous (S.C.) *KPC*2a tumors on day 14 posttumor. Each dot is an independent animal. Data are mean ± S.E.M. **p < 0.005, ***p < 0.0005, Student’s T test. **G** Representative IF for Cxcl9/Cxcl10 from normal mouse pancreas, *KPC*2 (CB-) parental tumors, *KPC*2a (CB +) tumors, or *KPC*2a tumors from mice treated with αPD-L1 on day 14. **H** Intensity units of Cxcl9/Cxcl10 + staining from samples in G. Each dot is an independent mouse. NP, normal pancreas. **I** Number of CD8 + T cells per field of view (FOV) from samples in G. **J** Correlation between CD8 + T cells and Cxcl9/Cxcl10 intensity from mice bearing *KPC*2a orthotopic tumors from **G**-**I**

As Cxcr3 is regulated on antigen-specific T cells during PDA growth (Fig. 1), we next tested if Cxcr3 deficiency impacted tumor growth and T cell infiltration. While spleens were significantly larger in *Cxcr3*^−/−^ vs. *Cxcr3*^+/+^ mice on day 21 post orthotopic tumor implantation, primary tumor weights were similar in *Cxcr3*^+/+^ and *Cxcr3*^−/−^ mice (Fig. 2A). CD8 T cell frequency was increased in *Cxcr3*^−/−^ spleens on day 21 (Fig. 2B), which correlated with increased spleen size (Fig. 2A). CD8 T cell frequency and number were similar in tumors from *Cxcr3*^−/−^ vs. *Cxcr3*^+/+^ mice at both timepoints (Fig. 2B). We next gated on tumor-specific T cells using the CB_101-109_:H-2D^b^ tetramer (Fig. 2C). In the spleen, we detected a trend for increased frequency of tumor-specific T cells in *Cxcr3*^−/−^ mice at day 21 (Fig. 2D), suggesting greater T cell retention, survival and/or proliferation. In PDA, tumor-specific T cell frequency and number were not significantly different among *Cxcr3*^+/+^ vs. *Cxcr3*^−/−^ mice at both timepoints (Fig. 2C–D). Immunofluorescent (IF) staining showed that CD8 T cells were infiltrating the tumor parenchyma in both *Cxcr3*^+/+^ and *Cxcr3*^−/−^ mice (Fig. 2E). Thus, Cxcr3 is dispensable for CD8 T cell infiltration into orthotopic PDA and had negligible impact on overall tumor control.

A role for Cxcr3 in T cell accumulation in tumors has been described in subcutaneous (s.c.) tumor implantation models [11–14]. Mechanisms governing T cell migration into skin tumors may be distinct from tumors that originate in internal organs and could explain why our results contrast with prior studies. *KPC*2a orthotopic tumors were five–tenfold larger than s.c. tumors in syngeneic C57Bl/6 J mice on day 14 (Fig. 2F), indicating that the tissue is a major determinant of pancreatic tumor growth. Spleens were also significantly larger in mice bearing orthotopic tumors vs. s.c. tumors (Fig. 2F), which likely reflects increased tumor burden as tumor cells produce factors that promote splenomegaly [32]. To test the hypothesis that Cxcr3 is critical for T cell migration into s.c. tumors, we implanted *KPC*2a tumor cells s.c. into *Cxcr3*^+/+^ or *Cxcr3*^−/−^ mice (Supplementary Fig. 2A). Tumor growth rate was similar early on, but by day 14, tumor volumes were modestly yet significantly reduced in *Cxcr3*^+/+^ mice compared to *Cxcr3*^−/−^ mice (Supplementary Fig. 2B–C). Tumor ulceration was delayed in *Cxcr3*^−/−^ mice (Supplementary Fig. 2D), which is consistent with a delay in tumor growth. Like our results in the orthotopic tumor model, the frequency and number of intratumoral CD8 T cells (Supplementary Fig. 2E) and tumor-specific CD8 T cells (Supplementary Fig. 2F) were similar in s.c. tumors from *Cxcr3*^+/+^ and *Cxcr3*^−/−^ mice on day 14. Thus, despite the accelerated antitumor response in *Cxcr3*^+/+^ mice bearing s.c. tumors, Cxcr3 was ultimately dispensable for tumor-specific T cell intratumoral accumulation in tumors growing within the skin. Together, the studies indicate tumor-specific T cells infiltrate PDA in a Cxcr3-independent manner.

Cxcr3 may not be required for antigen-specific T cell infiltration into PDA because ligands for Cxcr3, including Cxcl9 and Cxcl10 are absent in PDA. Therefore, we stained tumor sections for Cxcl9 and Cxcl10 from untreated *KPC*2a-bearing mice and mice treated with αPD-L1 or CD40 agonist as such therapies may enhance IFNγ [8, 28], which induces Cxcl9/Cxcl10. As expected, Cxcl9/Cxcl10 were mostly undetectable in normal mouse pancreas or parental *KPC*2 (CB-negative) orthotopic tumors on day 14 posttumor (Fig. 2G). In contrast, Cxcl9/Cxcl10 were modestly yet significantly increased in CB + *KPC*2a tumors and even further increased after αPD-L1 (Fig. 2G–H). CD8 T cells were increased in *KPC*2a tumors compared to normal pancreas and *KPC*2 tumors (Fig. 2I) and intratumoral CD8 T cell number correlated with Cxcl9/Cxcl10 in *KPC*2a tumors from control and αPD-L1-treated mice (Fig. 2J). CD40 agonist also increased Cxcl9/Cxcl10 in *KPC*2a tumors and Cxcl9/Cxcl10 colocalized with both tumor-associated macrophages (TAMs) and tumor cells (Supplementary Fig. 2G–H). Since we observed differences in Cxcl9/Cxcl10 staining in *KPC*2 vs. *KPC*2a tumors, we next tested if there were tumor cell intrinsic differences in chemokine expression by *KPC*2 and *KPC*2a cells. *KPC*2a cells expressed tenfold more *Cxcl9* and tenfold less *Cxcl10* as compared to the parental line in response to IFNγ (Supplementary Fig. 2I). However, IFNγ increased *Cxcl9* 4–5 logs and *Cxcl10* ~ 1–2 logs in both cell lines. These data are consistent with *Cxcl9* induction following IFNγ, whereas *Cxcl10* may be more sensitive to innate stimuli such as type I IFNs [9, 33]. Together, our results suggest that both antigenicity and immunotherapy increase Cxcl9/Cxcl10 in PDA.

### Cxcr3 alters T cell fate in a tissue-specific manner

**Fig. 3:**
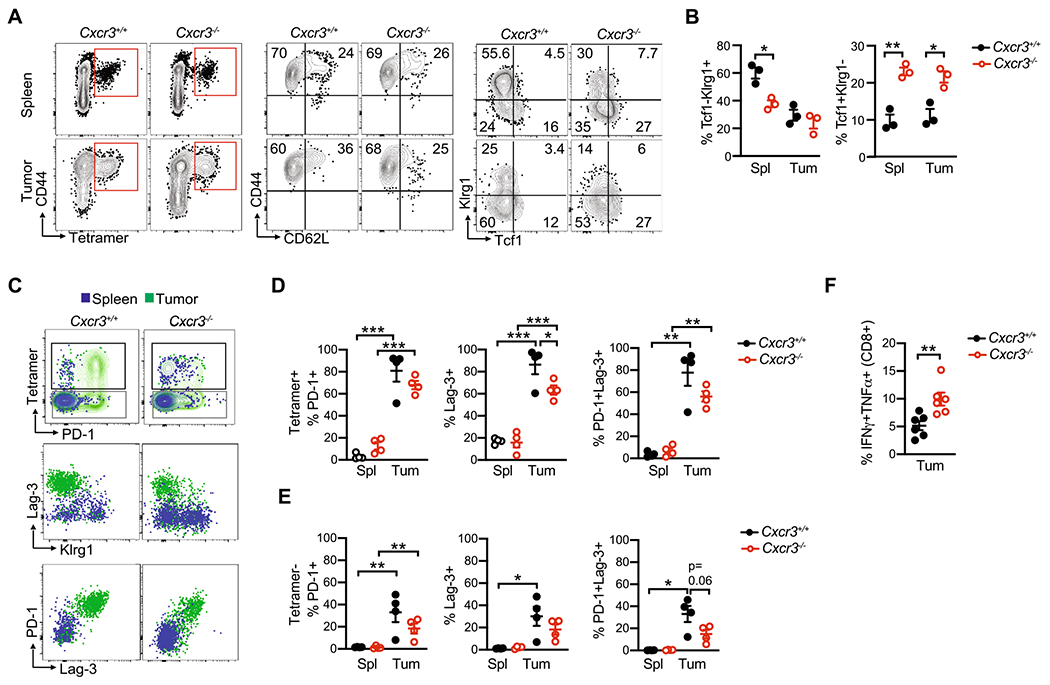
Cxcr3 alters T cell fate in a tissue-specific manner **A** Phenotype of CD8 + tetramer + T cells on day 14 post *KPC*2a orthotopic implantation into *Cxcr3*^+/+^ and *Cxcr3*^−/−^ mice. Flow cytometry plots show CD8 + tetramer + gates (left) for analyzing CD44, CD62L, Klrg1 and Tcf1. **B** Frequency of the indicated subpopulations from spleen and tumor gated on CD8 + tetramer + T cells 14 days following orthotopic *KPC2a* implantation. Data are mean ± S.E.M. Each dot is an independent animal. *p < 0.05, **p < 0.005. One-way ANOVA with a Tukey’s posttest. **C** Representative flow cytometric plots from spleen and tumor 14 days following orthotopic *KPC*2a implantation. Samples are gated on total CD8 + T cells (top row) to assess tetramer + cells and CD8 + tetramer + T cells (middle and bottom rows) to assess activation and exhaustion markers. **D** Proportion of CD8 + tetramer + T cells expressing the indicated receptors 14 days following orthotopic *KPC2a* implantation. Data are mean ± S.E.M. Each dot is an independent animal. *p < 0.05, **p < 0.05, ***p < 0.0005. ANOVA with a Tukey’s posttest. **E** Proportion of CD8 + tetramer− T cells expressing the indicated receptors 14 days following orthotopic *KPC2a* implantation. Data are mean ± S.E.M. Each dot is an independent animal. *p < 0.05, **p < 0.05, ***p < 0.0005. ANOVA with a Tukey’s posttest. **F** Proportion of total CD8 + T cells producing IFNγ and TNFα following a 5 h restimulation with CB_101-109_. Data are mean ± S.E.M. Each dot is an independent animal. **p < 0.005, Student’s T test

Cxcr3 promotes effector T cell differentiation at the expense of memory T cells during acute infection in mouse models [16–19]. Therefore, we hypothesized that Cxcr3 may influence tumor-specific T cell fate. We orthotopically implanted *KPC*2a tumor cells into the pancreas of *Cxcr3*^+/+^ and *Cxcr3*^−/−^ mice and analyzed CD8 + tetramer + T cell phenotype on day 14 posttumor. Using CD44 and CD62L markers to distinguish naïve, effector and memory T cells, most tetramer + T cells were a CD44 + CD62L− effector phenotype in both *Cxcr3*^+/+^ and *Cxcr3*^−/−^ mice (Fig. 3A). Klrg1 is expressed by effector T cells, whereas the memory/stem cell transcription factor Tcf1 is expressed by naïve and memory T cells. In both spleen and tumors from *Cxcr3*^−/−^ mice, tetramer + T cells had a higher frequency of Tcf1 + Klrg1− memory T cells and a lower frequency of Tcf1-Klrg1 + effector T cells (Fig. 3A–B). We previously showed that Klrg1 + Lag3− identifies functional effector T cells and Lag3 + Klrg1− identifies T_EX_ in *KPC*2a PDA [8, 25, 28]. In both *Cxcr3*^+/+^ and *Cxcr3*^−/−^ mice, tumor-infiltrating T cells upregulated PD-1 and Lag3 and lost Klrg1 as compared to splenic tetramer + T cells (Fig. 3C). The frequency of PD-1 + and/or Lag3 + tetramer + (Fig. 3D) and tetramer- (Fig. 3E) CD8 + T cells were significantly decreased in tumors from *Cxcr3*^−/−^ mice, suggesting Cxcr3 drives T_EX_. Supporting this interpretation, intratumoral *Cxcr3*^−/−^ tetramer + T cells exhibited significantly increased IFNγ production in response to CB_101-109_ peptide ex vivo (Fig. 3E). Thus, while Cxcr3 promoted effector T cell differentiation in the spleen, it paradoxically promoted T_EX_ differentiation in tumors.

### Cxcr3 sustains splenic Gzmb + antitumor T cells and mitigates cancer cell dissemination

**Fig. 4:**
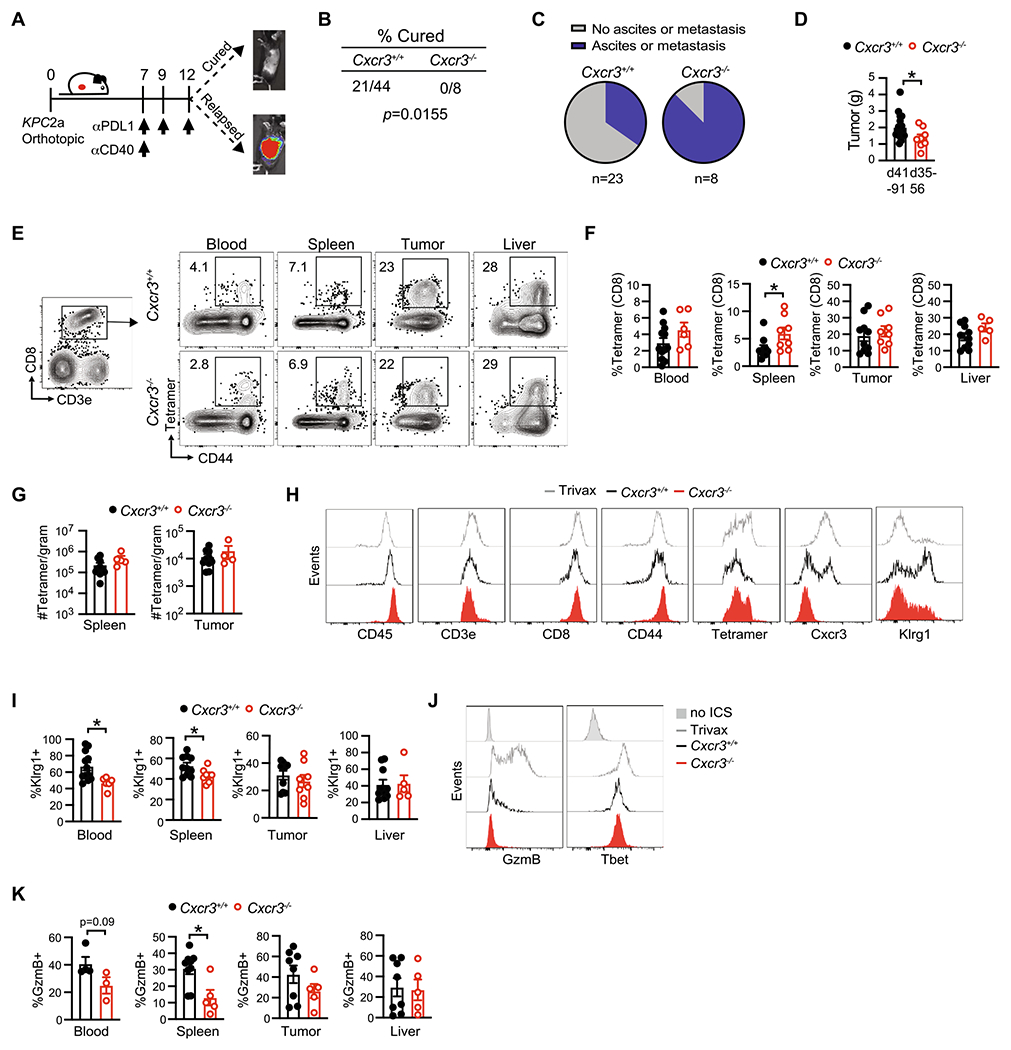
Cxcr3 sustains Klrg1 + Gzmb + antitumor T cells and mitigates cancer cell dissemination. **A** Simplified schematic for analysis of T cell persistence in mice that are cured or relapsed following immunotherapy. **B** Fraction of wild type and *Cxcr3*^−/−^ animals bearing *KPC*2a tumors that are cured following αPD-L1 + CD40 agonist by day 70 posttumor. Fisher’s exact test. Data are pooled from *n* = 2–4 independent experiments. **C** Proportion of relapsed mice that have metastasis and/or ascites at required euthanasia due to tumor burden 35–91 days post αPD-L1 + CD40 agonist. Data are pooled from *n* = 2–4 independent experiments. **D** Primary tumor weight (grams) at endpoint from relapsed mice in C. Each dot is an independent animal, *p < 0.05, Student’s T test. **E** Representative plots gated on CD8 + CD3 + T cells 35–91 days post αPD-Ll + CD40 agonist from relapsed *Cxcr3*^+/+^ or *Cxcr3*^−/−^ mice. Numbers in the plots are the percent tetramer +. **F** Frequency of tetramer + T cells of total CD8 + T cells. Data are mean ± S.E.M. Each dot is an independent animal. *p < 0.05, Student’s T test. **G** Number of CD8 + tetramer + T cells per gram of tissue from relapsed mice in D. Data are mean ± S.E.M. Each dot is an independent animal. **H** Representative histograms gated on splenic CD8 + tetramer + T cells from relapsed *Cxcr3*^+/+^ and *Cxcr3*^−/−^ mice. Splenic CD8 + tetramer + T cells from *Cxcr3*^+/+^ mice immunized with CD40 agonist + Poly:IC + CB_101-109_ peptide (Trivax) and assayed on day 7 post vaccination were included as a positive control. **I** Frequency of Klrg1 + tetramer CD8 + T cells in tissues from relapsed *Cxcr3*^+/+^ and *Cxcr3*^−/−^ mice. Data are mean ± S.E.M. Each dot is an independent animal. *p < 0.05, Student’s T test. **J** Representative histograms of intracellular GzmB and T-bet gated on splenic CD8 + tetramer + T cells from relapsed *Cxcr3*^+/+^ and *Cxcr3*^−/−^ mice. CD8 + tetramer + T cells without the intracellular staining (no ICS) served as a negative control. Splenic CD8 + tetramer + T cells from *Cxcr3*^+/+^ mice immunized with TriVax (CD40 agonist + Poly:IC + CB_101-109_ peptide) and assayed on day 7 post vaccination served as a positive control. **K** Frequency of GzmB + cells gated on tetramer + T cells in tissues from relapsed *Cxcr3*^+/+^ and *Cxcr3*^−/−^ mice. Data are mean ± S.E.M. Each dot is an independent animal. *p < 0.05, Student’s T test

We previously showed that αPD-L1 + CD40 agonist significantly prolonged survival of *KPC*2a-bearing mice [28]. In cured animals, tumor-specific T cells persist for over 100 days and reject orthotopic tumor rechallenge indicative of memory T cell formation [25, 26]. In non-cured mice, bioluminescent CB + tumors emerge indicative of relapse (Fig. 4A). Tumor-specific T cells persist indefinitely (up to at least ~ 125 days posttumor) and a fraction express Cxcr3 (Supplementary Fig. 3A). As Cxcr3 altered antitumor T cell fate (Fig. 3), we next tested if Cxcr3 played a role in the long-term durability of αPD-L1 + CD40 agonist therapy. By IVIS imaging of tumor growth of mice for up to 91 days posttumor, Cxcr3 loss impaired the long-term antitumor activity of αPD-L1 + CD40 agonist (Fig. 4B). We next compared tumor burden and metastasis in *Cxcr3*^+/+^ and *Cxcr3*^−/−^ mice that relapsed between 35–91 days posttumor. We observed increased ascites and/or metastasis in *Cxcr3*^−/−^ vs. *Cxcr3*^+/+^ relapsed mice (*p* = 0.0155, Fig. 4C). Primary tumor weights were smaller in *Cxcr3*^−/−^ vs. *Cxcr3*^+/+^ relapsed mice (Fig. 4D), indicating increased tumor dissemination in *Cxcr3*^−/−^ mice was not merely due to larger primary tumors and consistent with increased T cell IFNγ production at the earlier timepoint (Fig. 3F). To understand the basis for increased tumor cell dissemination in *Cxcr3*^−/−^ mice, we quantified tumor-specific T cells in relapsed *Cxcr3*^+/+^ and *Cxcr3*^−/−^ mice by tetramer staining. The frequency and number of tetramer + T cells (Fig. 4E–G) were similar between *Cxcr3*^+/+^ and *Cxcr3*^−/−^ mice, suggesting that increased tumor cell dissemination was not due to a deficit in maintaining tumor-specific T cell quantity. Overall CD8 + T cell frequency was also similar in multiple tissues from *Cxcr3*^+/+^ and *Cxcr3*^−/−^ relapsed mice (Supplementary Fig. 3B).We next evaluated effector T cell phenotype at these later timepoints, and included a positive control cohort of animals that were vaccinated with CD40 agonist + PolyIC + CB_101-109_ peptide [34]. The phenotype of intratumoral tetramer + T cells, including GzmB and T-bet, was similar in *Cxcr3*^−/−^ vs. *Cxcr3*^+/+^ mice (Supplementary Fig. 3C–D). In contrast, circulating and splenic tetramer + T cells from *Cxcr3*^−/−^ mice had decreased Klrg1 compared to tetramer + T cells from *Cxcr3*^+/+^ mice (Fig. 4I). Circulating and splenic tetramer + T cells from *Cxcr3*^−/−^ mice also exhibited decreased GzmB yet similar T-bet (Fig. 4J–K). These data suggest that the defect in Gzmb by *Cxcr3*^−/−^ T cells is not merely due to a global defect in the induction of the T-bet transcription factor.

### CD40 agonist promotes splenic Cxcl9 + *cDC1s and Cxcl9* + *Cxcl10* + *cDC2s*

**Fig. 5:**
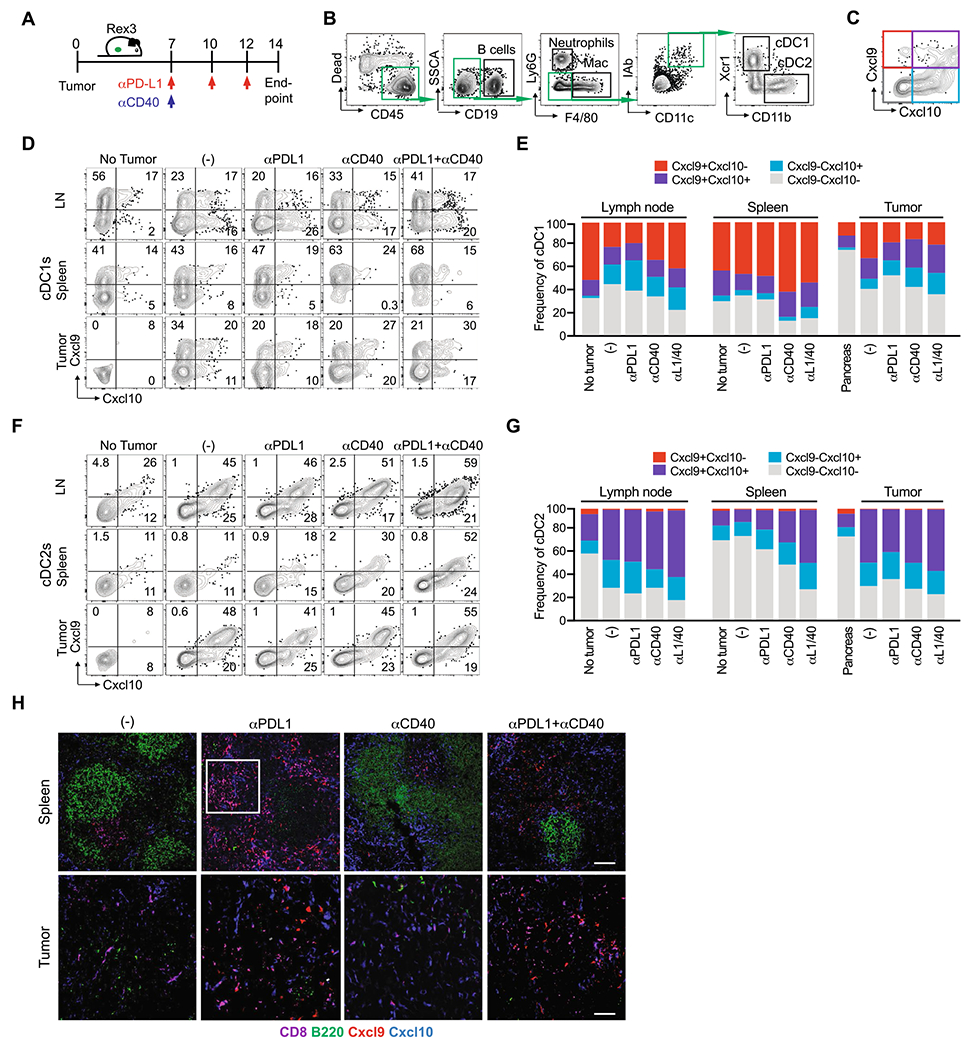
CD40 agonist increases Cxcl9 and Cxcl10 in a cell- and tissue-specific manner. **A** Schematic of immunotherapy in REX3 orthotopic *KPC*2a tumor-bearing mice. On day 7 post orthotopic tumor implantation, cohorts received isotype (−), αCD40 (100 μg), αPD-L1 (200 μg), or the combination as we described (28). αPD-L1 (200 μg) was also administered on day 10 and 12 posttumor for a total of 3 doses. **B** Flow cytometric gating strategy for analysis of indicated immune cells in spleen and tumor of mice depicted in (A). **C** Flow cytometric gating strategy for detecting Cxcl9 and/or Cxcl10 in non-tumor cells of REX3 mice depicted in (A). **D** Representative flow cytometry plots of cDC1s isolated from pancreatic draining lymph node (LN), spleen and tumor (or normal pancreas, *e.g.*, no tumor) on day 14 posttumor. **E** Mean frequency of cDC1s expressing Cxcl9 and/or Cxcl10. **F** Representative flow cytometry plots of cDC2s isolated from pancreatic draining LN, spleen, and tumor (or normal pancreas, *e.g.*, no tumor) on day 14 posttumor. **G** Mean frequency of cDC2s expressing Cxcl9 and/or Cxcl10. **H** Representative IF staining of CD8, B cells (B220) and Cxcl9 and Cxcl10 reporter expression in REX3 spleen and tumor from mice treated with the indicated therapies as in (A). Scale bar, 50 μm

A prior study suggested that conventional type 2 dendritic cells (cDC2s) in the spleen produce Cxcl9/cxcl10 in mice response to *T. gondii* chronic infection and may interact with Cxcr3-Klrg1 + T cells in the red pulp of the spleen [20]. Our prior study showed that conventional type 1 dendritic cells (cDC1s) were critical for both priming and maintenance of transferred effector or memory tumor-specific T cells in both the spleen and tumor [25]. To investigate the cellular source(s) of chemokine ligands in that may contribute to differentiation and/or maintenance of Klrg1 + GzmB + antitumor T cells in the spleen, we obtained the reporter of Cxcr3 ligands (REX3) mouse strain in which red fluorescent protein (RFP) reports *Cxcl9* and blue fluorescent protein (BFP) reports *Cxcl10* [19, 35]. We orthotopically implanted *KPC*2a tumor cells in REX3 mice and on day 7, administered αPD-L1, CD40 agonist or the combination (Fig. 5A) [28]. αPD-L1, CD40 agonist or the combination decreased tumor size and CD40 agonist increased spleen size in REX3 mice on day 14 posttumor (Supplementary Fig. 4A), similar to our prior study [28]. The proportion of immune subsets expressing Cxcl9 and/or Cxcl10 in the spleen, as well as the pancreatic draining lymph node (dLN) and tumor were assessed using the gating strategy in Fig. 5B–C. In non-tumor-bearing mice, 20–50% of cDC1s were producing Cxcl9 in the dLN, spleen and pancreas (Fig. 5D–E, Supplementary Fig. 4B). CD40 agonist significantly increased Cxcl9 + splenic cDC1s, whereas Cxcl10 was largely unchanged by this subset in the spleen (Fig. 5D–E, Supplementary Fig. 4B). In contrast, CD40 agonist promoted Cxcl10 by cDC1s in tumors (Fig. 5D–E, Supplementary Fig. 4B). Tumorigenesis decreased Cxcl9 + cDC1 frequency in the dLN and CD40 agonist reverted this phenotype (Fig. 5D–E, Supplementary Fig. 4B). Tumorigenesis increased the frequency of cDC2s that were producing Cxcl10 or Cxcl9 and Cxcl10 in the dLN and tumor, yet not the spleen (Fig. 5F–G, Supplementary Fig. 4B). However, CD40 agonist or the combination therapy significantly increased the frequency of Cxcl9 + Cxcl10 + cDC2s in the spleen (Fig. 5F–G, Supplementary Fig. 4B). Thus, these data reveal that both tumorigenesis and CD40 agonist alters Cxcl9/Cxcl10 production by DCs in a tissue-specific manner. IF staining confirmed an abundance of Cxcl10 + and Cxcl9 + cells primarily located in the spleen red pulp following immunotherapy (Fig. 5H). We noted an expansion of CD8 + T cells in the red pulp in the spleen following αPD-L1 or αPD-L1 + CD40 agonist, which is consistent with an expansion of splenic CD8 + tetramer + T cells following PD-L1 blockade [8, 28]. In contrast, tumor tissue staining by IF showed predominantly Cxcl10 + cells (Supplementary Fig. 4C). As we showed previously [25], tumors are densely populated by macrophages and granulocytes and these cells are producing Cxcl10 rather than Cxcl9 (Supplementary Fig. 4D–F). The differential expression of Cxcl10 and Cxcl9 in tumor vs. spleen likely reflects different composition of myeloid cells and raises the possibility that the chemokines may play a differential role in the T_EFF_ vs. T_EX_ fate choice. However, further studies will be necessary to specifically identify the distinct contribution of DC subset-derived chemokines on antitumor T cell differentiation.

### Subcellular Cxcr3 ligand distribution is altered by immunotherapy and Cxcr3 promotes GzmB + engineered T cells that mitigate metastasis

**Fig. 6:**
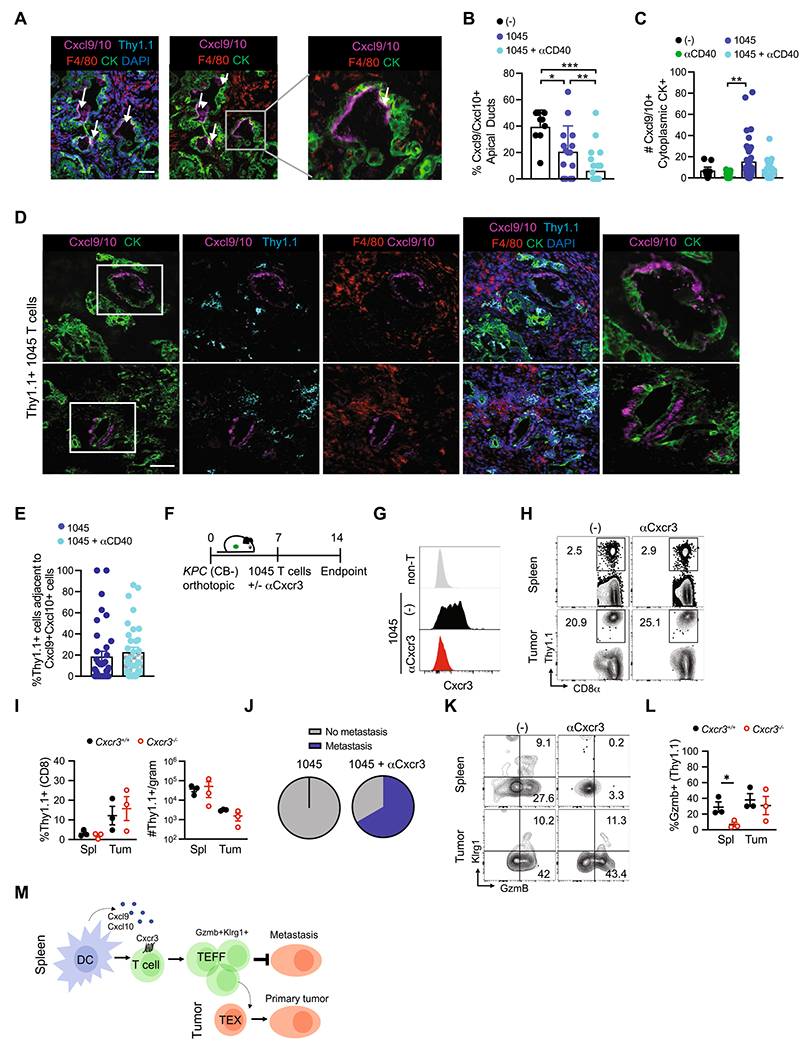
Subcellular Cxcr3 ligand distribution is altered by immunotherapy and Cxcr3 promotes GzmB + engineered T cells that mitigate metastasis. **A** Apical Cxcl9/10 by cytokeratin + (CK) ductal tumor cells from *KPC* GEMM tumors from untreated mice. Scale bar, 50 μm. **B** Percentage of CK + tumor cells that express apical Cxcl9/Cxc10. *p<0.05, **p<0.005, ***p<0.0005, one-way ANOVA with a Tukey’s post test. **C** Number of tumor cells that express cytoplasmic Cxcl9/Cxc10 following immunotherapy. **p<0.005, one-way ANOVA with a Tukey’s post test. **D** Cxcl9/Cxcl10 in *KPC* GEMM tumors on day 8 post Thy1.1 + 1045 T cells. Scale bar, 50 μm. **D** Representative IF of tumor sections from *KPC* mice treated with 1045 T cell therapy (1045 T cells) at 8 days post treatment. IF overlays of Cxcl9/Cxcl10, Thy1.1 expressed on engineered T cells, macrophage marker F4/80, tumor cell marker cytokeratin (CK) and DAPI nuclear stain. **E** Frequency of Thy1.1 + engineered T cells adjacent to Cxcl9/Cxc10 + cells in PDA from tissues depicted in D. **F** Experimental schematic for mice receiving orthotopic *KPC2* (CB-) PDA cells, adoptive transfer of 1045 Thy1.1 + T cells and anti-Cxcr3 treatment days 6, 9 and 12, or untreated controls. **G** Representative histograms of Cxcr3 staining by adoptively transferred 1045 T cells in untreated (−) and anti-Cxcr3 treated mice compared to non-T cells (top panel) in mice depicted in **F**. **H** Proportion of splenic and intratumoral transferred Thy1.1 + CD8 + T cells in untreated (−) and anti-Cxcr3 treated mice depicted in **F**. **I** Proportion of total CD8 + T cells and total Thy1.1 + cell numbers per gram tissue of mice depicted in **F**. **J** Proportion of mice depicted in **F** which developed metastasis, *n* = 3 mice per group. **K** Representative flow cytometric plots of Klrg1 and Granzyme B (GzmB) staining on splenic and intratumoral Thy1.1 + CD8 + adoptively transferred 1045 CD8 T cells isolated from untreated (−) or anti-Cxcr3 treated mice 2 weeks following orthotopic tumor implantation. **L** Proportion of splenic and intratumoral Granzyme B + adoptively transferred Thy1.1 + CD8 T cells 2 weeks following orthotopic tumor implantation. *n* = 3 mice per group, significance determined by Student’s T test. *, *p*<0.05. **M** Simplified model depicting the role for Cxcr3 in promoting effector T cell differentiation in the spleen and exhausted T cell differentiation in the tumor microenvironment

CB is a model antigen and the CB_101-109_ binding affinity to H-2D^b^ is predicted to be quite high [8], thereby modeling a particularly avid antitumor T cell response. Therefore, we next asked if Cxcr3 played a role in limiting metastasis during adoptive T cell therapy using mesothelin (Msln_406-414_:H-2D^b^)-specific 1045 TCR engineered T cells which we previously showed have therapeutic efficacy in the autochthonous *KPC* GEMM [7]. We first analyzed Cxcl9/Cxcl10 distribution in tumors from unmanipulated *KPC* GEMM. Unexpectedly, Cxcl9/Cxcl10 was largely confined to the apical surface in tumor cells from untreated *KPC* mice (Fig. 6A). The frequency of tumor cells expressing apical Cxcl9/Cxcl10 was significantly decreased in *KPC* recipients of 1045 T cells or 1045 T cells + CD40 agonist (Fig. 6B). Instead, engineered T cells + CD40 agonist, which we previously showed to promote the persistence of engineered T cells in autochthonous PDA [36], promoted tumor cytoplasmic Cxcl9/Cxcl10 (Fig. 6C–D, Supplementary Fig. 5A), indicating that immunotherapy alters the subcellular localization of Cxcr3 ligands in tumor cells. These data suggest that immunotherapy may result in tumor cell chemokine secretion into the parenchyma rather than into the ductal space, thereby orchestrating the intratumoral migration patterns of Cxcr3 + cells within the stroma. Since 1045 TCR engineered T cells express Cxcr3 prior to transfer (Supplementary Fig. 5B), we next quantified the frequency of Thy1.1 + 1045 T cells adjacent to Cxcl9/Cxcl10 + cells. We observed substantial variability in the frequency of Thy1.1 + T cells adjacent to Cxcl9/Cxcl10 + cells in PDA (Fig. 6E), suggesting that Cxcr3-independent factors influence effector T cell migration within the stroma. In stromal-rich regions that lacked CK + tumor cells, 1045 T cells + CD40 agonist increased Cxcl9/Cxcl10 expression by macrophage and 1045 T cells appeared to colocalize with the Cxcl9 + macrophage in these stromal regions (Supplementary Fig. 5C), similar to our results in resected human PDA [37]. Together, these data support that engineered T cell therapy alters Cxcl9/Cxcl10 spatial, cellular and subcellular localization in autochthonous PDA.

To assess the requirement for Cxcr3 on 1045 T cell infiltration into PDA, 1045 T cells were transferred into mice bearing established and poorly immunogenic (CB-) *KPC*2 orthotopic tumors [8] using a similar therapy protocol that has efficacy in autochthonous *KPC* PDA [7] with or without anti-Cxcr3 (Fig. 6F). As expected, anti-Cxcr3 interfered with Cxcr3 on engineered T cells (Fig. 6G). Consistent with our studies of endogenous CB-specific T cells, Cxcr3 blockade did not impact 1045 T cell accumulation in PDA (Fig. 6H–I). However, Cxcr3 blockade resulted in more mice with detectable macrometastasis following engineered T cell therapy on day 14 (*p* = 0.0533, Fig. 6J). Notably, Cxcr3 blockade interfered with the frequency of splenic engineered T cells that expressed Granzyme B (GzmB) (Fig. 6K–L), paralleling the defect in endogenous GzmB + tumor-specific T cells in *Cxcr3*^−/−^ mice (Fig. 4K). Together, our results support that Cxcr3, potentially by interacting with Cxcl9/Cxc10 + cDC1s in the spleen, promotes GzmB + cytotoxic T cells that may mitigate pancreatic cancer metastasis (Fig. 6M). Since Cxcr3 also promotes T_EX_ in primary tumors (Figs. 3D–F and 6M) the data support a tissue-specific role for Cxcr3 on T cell fate during malignancy.

## Discussion

Here, we identify paradoxical roles for Cxcr3 on tumor antigen-specific T cell fate and antitumor functionality during PDA progression and immunotherapy response. Cxcr3 sustained splenic Klrg1 + Gzmb + antitumor T cells that may aid in the control of disseminated disease. Thus, Cxcr3, potentially through positioning of T cells in secondary lymphoid organs [19], promotes cytotoxic T cells likely capable of targeting metastatic tumor cells. Unexpectedly, we identify that Cxcr3 was dispensable for intratumoral T cell accumulation and instead promoted T_EX_ differentiation in primary tumors. Therefore, tissue-specific modulation of Cxcr3 may provide a novel therapeutic axis to uncouple T_EX_ from T_EFF_ differentiation.

Prior studies in other chronic antigen settings have identified rare PD-1 + Tcf1 + progenitor T cells that mediate response to PD-L1 blockade [38–40]. Since PD-1 is not clearly expressed on most splenic tumor-specific T cells (Fig. 1) [8, 25, 28], and migration of T cells from periphery into tumors is required for αPD-L1 transient antitumor activity [8], we sought other markers to identify peripheral tumor-specific T cell subsets that mediate immunotherapy response. During persistent *T. gondii* infection in mice, three subsets of splenic antigen-specific T cells were identified based on Cxcr3 and/or Klrg1 [29]. The Cxcr3 + Klrg1− subset exhibited the greatest proliferative capacity and seeded an intermediate Cxcr3+ Klrg1 + subset with memory and effector cell traits that gave rise to the cytotoxic terminally differentiated Cxcr3-Klrg1 + subset [29]. Here, we show in the spleen of tumor-bearing mice early on during tumor establishment, most tetramer + T cells expressed Cxcr3 and/or Klrg1 and the intermediate Cxcr3+Klrg1 + subpopulation in the spleen is particularly enriched for tumor antigen specificity. Over time, Cxcr3 and Klrg1 were progressively decreased on tumor-specific T cells infiltrating PDA and these Cxcr3-Klrg1− T cells co-expressed PD-1 and Lag-3, markers that identify T_EX_ cells defective in IFNγ production [8]. Cxcr3 and Klrg1 are also downregulated by T_EX_ during chronic viral infection [41] and Cxcr3 is downregulated on T cells after multiple rounds of antigen exposure in vivo [42], suggesting that Cxcr3 loss in PDA is due to persistent TCR signaling. At least at the gene level, three CD8 + T cell clusters infiltrating human PDA expressed *CXCR3* and variable levels of *KLRG1*, suggesting a similar pattern in human disease. However, additional studies are needed to determine the extent such markers can be utilized to map human CD8 T cell differentiation states. In human PDA, *PDCD1* + *LAG3* + were among *KLRG1* + CD8 T cell clusters consistent with a connection between exhausted and effector T cell states [43].

We previously showed that Batf3 + Xcr1 + cDC1s are required to not only prime tumor-specific CD8 T cells in the spleen, but also maintain adoptively transferred Klrg1 + effector T cells in both spleen and tumor [25]. Our prior study also indicated that cDC1s promote Lag3 + T_EX_ in the PDA [25], paralleling our findings here that show Cxcr3 can promote Klrg1 + T cells in the spleen and Lag3 + T_EX_ in primary tumors. As we find that cDC1s uniquely express Cxcl9 without Cxcl10, the Cxcl9-Cxcr3 axis may be responsible for the tissue-specific outcomes on cytotoxic T cell fate. This chemokine axis may promote T cell:cDC1 interactions that impact T cell differentiation in a tissue-specific manner. In a prior study, Cxcl9 but not Cxcl10 expression by cDC1s was critical for αPD-L1 efficacy which was attributed to intratumoral T cell trafficking toward cDC1s in the tumor bed in s.c. implantable tumor models [13]. We find that cDC1s are poised to produce Cxcl9, whereas more abundant intratumoral myeloid cells including macrophages, cDC2s and granulocytes [25] are biased toward Cxcl10 production. A prior study suggested that cDC2s, which reside in the bridging channels in the spleen, sustains Klrg1 + pathogen-specific T cells during chronic infection [20]. As cDCls and cDC2s contribute to antitumor immunity in part through induction of tumor-reactive CD8 + and CD4 + T cells, respectively, further investigation into the specific contribution of these cells on T cell differentiation during tumor growth and immunotherapy is of interest [44]. Additional factors beyond antigen presentation and Cxcl9/Cxcl10 that DCs are known to express including IL-15 [45] or IL-12 [46] may also be involved in impacting T cell differentiation and maintenance.

We show here that Cxcr3 is not a principal mechanism governing T cell migration into PDA and raise the possibility that other chemokine receptors may play a role in guiding antitumor T cells into the tumor bed. Alternatively, cognate antigen presentation by APCs or graft endothelial cells was sufficient for antigen-specific T cell migration in a heart allograft model [47], whereas the migration of non-specific bystander T cells remained G-protein signaling dependent [47]. Cxcr3 was shown to promote CD8 T cell migration in B16 melanoma [11, 48] and other s.c tumor models [49]. Since we observed a subtle delay in antitumor effects in *Cxcr3*^−/−^ mice bearing s.c. tumors, our results suggest a potential role for Cxcr3 in the initial wave of T cell migration into the skin.

Cxcr3 promoted the differentiation of Klrg1 + PDA-specific T cells in the spleen of tumor-bearing mice, consistent with Cxcr3 promoting short-lived effector T cells in acute infection models [16–19]. Cxcr3 guides T cells to LN periphery to support effector T cell differentiation during infection [19]. During persistent *T. gondii* infection, Cxcr3 loss led to an expanded Ki67 + Klrg1− memory T cells defective in differentiating into Klrg1 + T cells [20]. Klrg1 can mark effector T cells that localize in the vasculature, including the red pulp of the spleen, which promotes enhanced capacity to clear systemic infections [50, 51]. Our results raise the possibility that this localization, in addition to cytotoxic functionality, may poise such T cells to rapidly respond to circulating metastatic cancer cells. In sum, our results support a role for Cxcr3 in the spleen to promote the differentiation of potent Gzmb + antitumor T cells that may aid in the control of tumor cell dissemination. As metastasis is a leading cause of cancer-related mortality, further investigation into how to generate and maintain such cells could inform superior T cell-based immunotherapies for cancer patient treatment.

## Materials and methods

### Animals

*Kras*^LSLG12D/+^;*Trp53*^LSLR172H/+^;*p48*Cre (*KPC*) mice previously speed-backcrossed to > 99.6% genetic similarity to C57BL/6 J mice were used [7]. 6- to 12-wk-old female and male C57BL/6 J mice were purchased from The Jackson Laboratory (000,664). C57BL/6 J backcrossed *Cxcr3*^−/−^ mice were generously provided by Dr. Sara Hamilton (University of Minnesota). REX3 transgenic mice [35] were kindly provided by Dr. Andrew Luster (Massachusetts General Hospital) and obtained from Dr. Marc Jenkins (University of Minnesota). University of Minnesota Institutional Animal Care and Use Committee approved all animal studies.

### Tumor cell lines

We previously described the *KPC*2 parental and the *KPC*2a cell line retrovirally transduced to express click-beetle red luciferase linked to eGFP (CB-eGFP) [8]. Tumor cells were cultured in Basic media: DMEM (Life Technologies) + 10% FBS (Life Technologies) + 2.5 mg/ml amphotericin B (Life Technologies) + 100 mg/ml penicillin/streptomycin (Life Technologies) + 2.5 mg dextrose (Fisher Chemical) at 37 C + 5% CO_2_. Media was sterile filtered and stored in the dark at 4 C. Cell lines used for experiments were maintained below passage 15. 0.25% trypsin–EDTA (Thermo Fisher) was used for serial passages.

### Tumor cell implantation

For orthotopic tumor implantation, mice received slow-release buprenorphine injected subcutaneously prior to surgery for analgesia according to approved protocols. Mice were anesthetized using continuous flow of 2–5% isoflurane. Hair was removed using clippers followed by Nair. The abdomen was sterilized using a series of EtOH and Betadine washes. Once mice reached surgical plane anesthesia, a small incision was made in the abdomen followed by a small incision in the peritoneum to access the pancreas. 1 × 10^5^ *KPC*2a cells in 20 μl of 60% Matrigel were injected into the pancreas using an insulin syringe (Covidien) as we described [8, 28]. Separate sets of sutures were used to close the peritoneum and skin (Ethicon). For subcutaneous (s.c.) tumor implantation, 50 μl containing 1 × 10^6^ *KPC*2a cells in of 60% Matrigel (Discovery Labware) was injected above the hind flank. Tumor volume was measured using calipers.

### In vivo antibody treatments

Seven days post orthotopic tumor implantation, a single 100 μg dose of agonistic αCD40 (FGK45, BioXcell) was administered alone or in combination with 200 μg αPD-L1 (10F.9G2, BioXcell) on days 7, 10, and 12 i.p. [28]. 200 μg of αCxcr3 (CXCR-173, BioXcell) was administered on days 6, 9 and 12.

### Bioluminescent tumor imaging in vivo

Abdominal hair was removed with Nair. Mice were injected i.p. with 100 μg of D-Luciferin (Promega). Six–10 min after D-Luciferin injection, images were acquired after 0.5 s exposure time with a binning of 8 as we described [8, 28]. As luminescence saturation may occur, additional images with a binning of 2 and/or auto exposure setting were acquired. Tumor radiance was quantified in photons per second using IVIS 100 and Living Image software (Xenogen).

### Preparation of mononuclear cells from tissues

Spleens were mechanically dissociated to single cells followed by red blood cell (RBC) lysis in 1 ml of Tris-ammonium chloride (ACK) lysis buffer (Life Technologies) for 2 min at rt. RBC lysis was quenched by addition of 9 ml of T cell media. Splenocytes were spun at 1400 rpm for 5 min and kept on ice. Tumors were mechanically digested to single-cell suspensions, collagenase digested and washed 2X to remove cell debris and pancreatic enzymes. Peripheral blood was collected in Eppendorf tubes containing 20 μL of 20 mM EDTA. PBMCs were spun for 8 min at 10,000 rpm at 4 °C and plasma was removed. RBCs were lysed with 1 ml of ACK Lysis buffer (GIBCO) for 8 min at rt. If incomplete lysis occurred, cells were spun down for a second time at 13,000 rpm for 30 s and the ACK lysis step was repeated.

### Real-time PCR

*KPC2* and *KPC*2a cells were cultured in vitro ± recombinant murine IFNγ (100 ng/mL, R&D Systems). After 24 h, RNA was extracted using the RNeasy Mini Kit (Qiagen) and concentration and purity of RNA was determined by Nanodrop. cDNA was generated using RT Buffer Mix and RT Enzyme Mix (Thermo Fisher). Real-time PCR was performed in triplicate on a BioRad CFX96 Touch Real-Time PCR Detection System by measuring SYBR Green fluorescence in triplicate for 40 cycles. ATP5b was the housekeeping gene. Relative quantification was determined using the delta-delta Ct method, also known as the 2^−ΔΔCt^ method [52]. Fold gene expression was calculated using 2^-(ΔΔCt) where ΔCt=Ct (gene of interest) – Ct (housekeeping gene) and ΔΔCt = ΔCt (Sample) – ΔCt (control average).

### Cell surface staining

Cells were stained in the presence of 1:500 Fc block (αCD16/32, Tonbo). CB_101-109_:H-2D^b^-BV421 tetramer was generated in laboratory [8, 28] and diluted 1:100 in FACs buffer (PBS + 2.5% FBS). For analysis of immune cells, fluorescently conjugated monoclonal antibodies were diluted 1:100 in FACs buffer and include CD45 (30F-11, Biolegend), CD3e (17A2, Biolegend), CD8 α(53–6.7, Tonbo), CD44 (IM7, BD), CD4 (RM4.5, Tonbo), Klrg1 (2F1, Biolegend), Cxcr3 (Cxcr3-173, Biolegend), PD-1 (J43, Invitrogen), Lag-3 (C9B7W, Biolegend), CD69 (H1.2F3, BD), CD62L (Mel14, Biolegend), CD11b (M1/70, Tonbo), CD19 (1D3, BD), NK1.1 (PK136, eBioscience), Ly6G (1A8, eBioscience), F4/80 (T45-2342, BD), CD11c (N418, BD), I-A^b^ (M5/114 15.2, Invitrogen), Ly6C (HK1.4, eBioscience) and Xcr1 (ZET, Biolegend). Cells were stained for 30 min at 4 °C in the dark and washed 2X with FACs buffer. Viability dyes BV510 or APCe780 (Tonbo, 1:500) were used to exclude dead cells and cell counting beads (Sigma-Aldrich) were added prior to analysis to calculate cell number per tissue gram. Cells were fixed in 2% PFA or fixation buffer (Tonbo) for 10–15 min at 4 °C in the dark prior to data acquisition. For detecting REX3 reporters, cells were not fixed and acquired immediately after staining. Cell analysis was performed using FlowJo software (version 10). Cells were acquired within 24 h using a Fortessa 1770 and data analyzed using FACS Diva software (BD).

### Intracellular staining

The Foxp3 intracellular staining kit (Tonbo) was used for detecting intracellular proteins. Following cell surface staining, cells were washed 2X in FACs buffer, fixed for 30 min at 4 °C, washed 2X in permeabilization buffer and stained with Gzmb (NGZB, eBiosciences), Tcf1 (S33-966, BD), Foxp3 (FJK-16 s, eBioscience) and Helios (22F6, BD). Antibodies were diluted 1:100 in permeabilization buffer and cells were stained 1–2 h in the dark at 4 °C. Cells were washed 2X in permeabilization buffer and acquired within 24 h on a Fortessa 1770 flow cytometer following addition of cell counting beads (Sigma). Cell analysis was performed using FlowJo software (version 10).

### Engineered T cell therapy

DMEM (Life Technologies) + 10% FBS (Life Technologies), 100 μg/ml penicillin/streptomycin (Life Technologies), 20 mM l-glutamine (Life Technologies), 1X NEAA (Life Technologies) and 50 μM 2-betamercaptoethanal (Sigma-Aldrich) were used to culture T cells in vitro. Mononuclear cells from spleens of P14 Thy1.1 + mice were stimulated in vitro with 1 mg/mL of αCD3 (145-2C11, BD) + 1 mg/mL αCD28 (37.51, BD) in 10 mL of T cell media containing human recombinant IL-2 (rIL2, 50 U/mL, Peprotech) in T25 flasks at 37 °C and 5% CO2 as described [7, 36]. On days 1 and 2 after stimulation, activated T cells were transduced with retroviral supernatant containing the 1045 TCR [7] by spinfection in 12-well plates containing polybrene (10 mg/mL) and rIL2 (50 U/mL) for 90 min at 1,000 × g at 32 °C. On day 7, T cells were restimulated with Msln_406-414_ (GQKMNAQAI) pulsed irradiated splenocytes and rIL2. Five days post the 2^nd^ stim, T cells were injected i.p. into mice as we previously described [7, 36]. At the time of T cell transfer, recipients either received 100 μg i.p. of isotype or agonistic αCD40 (FGK45, BioXcell) as described [36]. For cell therapy experiments in Fig. 6, B6 mice were orthotopically implanted with 1 × 10^5^ parental CB-negative *KPC2* tumor cells [8]. Donor Thy1.1 + T cells expressing the high affinity Msln_406-414_:H-2D^b^-restricted TCR (clone 1045) were isolated from the spleens of 1045 TCR *Trac* knockin mice (Rollins *et. al., in revision*) for studies in Fig. 6. T cells were transferred 6 h post Cytoxan (180 mg/kg, Amneal Pharmaceuticals) on day 6 posttumor ± 200 μg anti-Cxcr3 (Cxcr3-173, BioXcell) i.p. on days 6, 9 and 12. Recipients received rhIL-2 10^4^ U i.p. on day 6, 8, 10 and Msln_406-414_ pulsed irradiated splenocytes on day 6 to support donor T cell expansion similar to as described [7, 36].

### Immunofluorescence

Tissues were embedded in OCT (Tissue-Tek) and stored at − 80 °C. 7 μm sections were cut using a Cryostat and fixed in acetone at − 20 °C for 10 min. Sections were rehydrated with PBS + 1% bovine serum albumin (BSA) and incubated for 1 h at room temperature (rt) with 1:200 αCD8α-PerCp-Cy5.5 (53–6.7, BD), 1:500 αThy 1.1-BV510 (OX-7, Biolegend), 1:200 αF4/80-PE (BM8, Biolegend), 1:200 αpanCK-FITC (F3418, Sigma-Aldrich), 1:100 Cxcl9-EF660 (MIG-2F5.5, eBioscience) and 1:100 αCxc110 (goat polyclonal, R&D Systems) diluted in PBS + 1% BSA. Slides were washed 3X in PBS + 1% BSA and incubated with anti-goat 1:500 AF647 (Jackson ImmunoResearch, for detecting αCXCL10) for 1 h at rt in the dark. Cxcl9 EF660 and Cxcl10 AF647 were detected in the APC channel. Tissues were washed 3X with PBS + 1% BSA, washed 3X with PBS, and mounted in DAPI Prolong Gold (Life Technologies). Images were acquired on a Leica DM6000 epifluorescent microscope at the University of Minnesota Center for Immunology using Imaris 9.1.0 (Bitplane). To determine CD8 T cell number, individual cells from *n* = 3 mice per group and a minimum of 3–10 fields per section were manually counted and recorded by an investigator blinded to the experimental conditions using Cell Counter in Fiji2.0. Cxcl9/10 staining intensity was measured by pixel intensity from 3 mice per group and 3–8 fields per section using Fiji2.0. In tissues from REX3 mice, Cxcl9 (PE) and Cxcl10 (BFP) reporters were detected ex vivo in 7 μm OCT-embedded sections. These sections were also stained with αCD8 (53–6.7, BD) and αB220 (RA3-6B2, Biolegend) diluted 1:100 in PBS + 1%BSA for 1 h in the dark, followed by washing 3X as above. These images were acquired on a Leica DM4 B microscope. Images were analyzed using Fiji2.0.

### scRNAseq analysis

Filtered count matrices for 9 human samples from *Elyada* et al. study [30] were obtained after NIH approval at dbGaP (accession number phs001840.v1.p1) and were used as input data. Barcodes that were considered to represent noise and poor-quality cells were removed using the knee-inflection strategy implemented in DropletUtils package (version 1.10.3). For downstream analysis, Seurat package (version 3.1.0) was used. Genes expressed in fewer than 3 cells were filtered from the expression matrices as well as cells that expressed fewer than 200 genes. Finally, cells with a mitochondrial percentage more than the highest confidence interval for scaled mitochondrial content were filtered out. Each sample was normalized using the *SCTransform* function with mitochondrial content as a variable to regress out in a second non-regularized linear regression. Before integration, highly variable features across the samples were found by *SelectIntegrationFeatures* function with the number of genes equal to 2000, then the object was prepared for integration (*PrepSCTIntegration* function), the anchors were found (*FindIntegrationAnchors* function), and the samples were integrated into the whole object (*IntegrateData* function). Principal component analysis was applied for dimensionality reduction, and the first 20 principal components were used further to generate uniform manifold approximation and projection (UMAP) dimensionality reduction. The clustering procedure was performed by *FindNeighbors* and *FindClusters* (resolution 0.8). After that, cluster 7 enriched by low-quality cells was removed, and only 6 tumor samples (SRR9274536, SRR9274537, SRR9274538, SRR9274539, SRR9274542, SRR9274544) were preserved. Remaining cells were fully renormalized, reintegrated and re-clustered.

### Statistical analysis

Statistical analyses were performed using GraphPad software (version 9.0). Mouse experiments include = 3–23 mice per group. Unpaired, two-tailed Student’s T test was used to compare two-group data. One-way ANOVA and Tukey posttest were used for comparing > 2-group data. Log-rank (Mantel–Cox) test was used to test for statistically significant differences in survival. Data are presented as mean ± standard error of the mean (SEM), and *p* < 0.05 was considered significant. *p < 0.05, **p < 0.005 and ***p < 0.0005.

## Supplementary Material

Supplementary Figures
