# Supplementary material for: Cxcr3 constrains pancreatic cancer dissemination through instructing T cell fate
Source: Cancer Immunol Immunother. Author manuscript; Available in PMC 2023 Jun 1. (PMC10198906; doi:10.1007/s00262-022-03338-7)
Supplement: Supplementary file 1 [file NIHMS1871049-supplement-Supplementary_Figures.pdf]

Supplementary Figures 1-5

Burrack *et. al*. 2022

Supplementary Figure 1

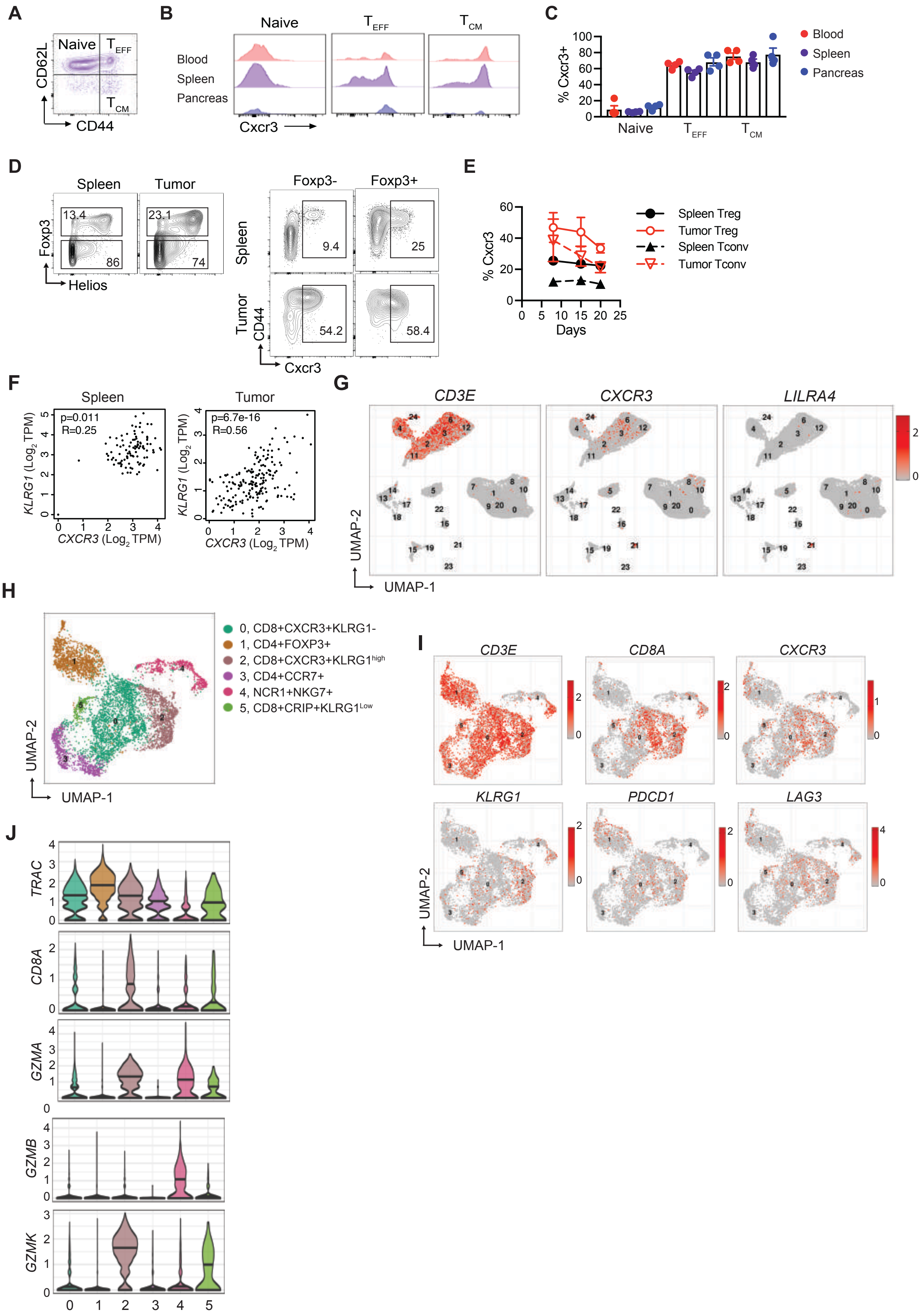

Supplementary Figure 1. Cxcr3 is expressed by T cells in human and murine PDA. A) Representative CD62L and CD44 staining on circulating CD8+ T cells from naïve B6 mice to distinguish naïve, effector (Teff), and central memory (T_{CM}) T cell populations. **B**) Histogram of Cxcr3 gated on the indicated CD8 T cell subsets. **C**) Frequency of Cxcr3+ cells from each CD8 T cell subset. Each dot is an independent animal. Data are mean ± S.E.M. n=4 mice per group. **D**) Gating strategy for Treg among splenic and intratumoral CD4+CD44+ T cells (left) for analysis of Cxcr3. **E**) Proportion Cxcr3+ Foxp3+ (Treg) and Foxp3- (Tconv) CD4 T cells over time in spleen and tumor. n=4-5 mice per group. Data are mean ± S.E.M. n=4 mice per group. **F**) Log_2 *KLRG1* and *CXCR3* from spleen and tumor from human PDA patients were obtained from TCGA and analyzed using GEPIA. **G**) Normalized log2 expression of the indicated genes in 6 merged human PDAs from the *Elyada et. al*. study. **H**) UMAP of re-analyzed *CD3E*+ cells from G. Clusters were manually named. **I**) Normalized log2 expression of the indicated genes among T cell clusters in H. **J**) Violin plots (showing normalized log2 expression) from the 5 lymphocyte clusters in H.

Supplementary Figure 2

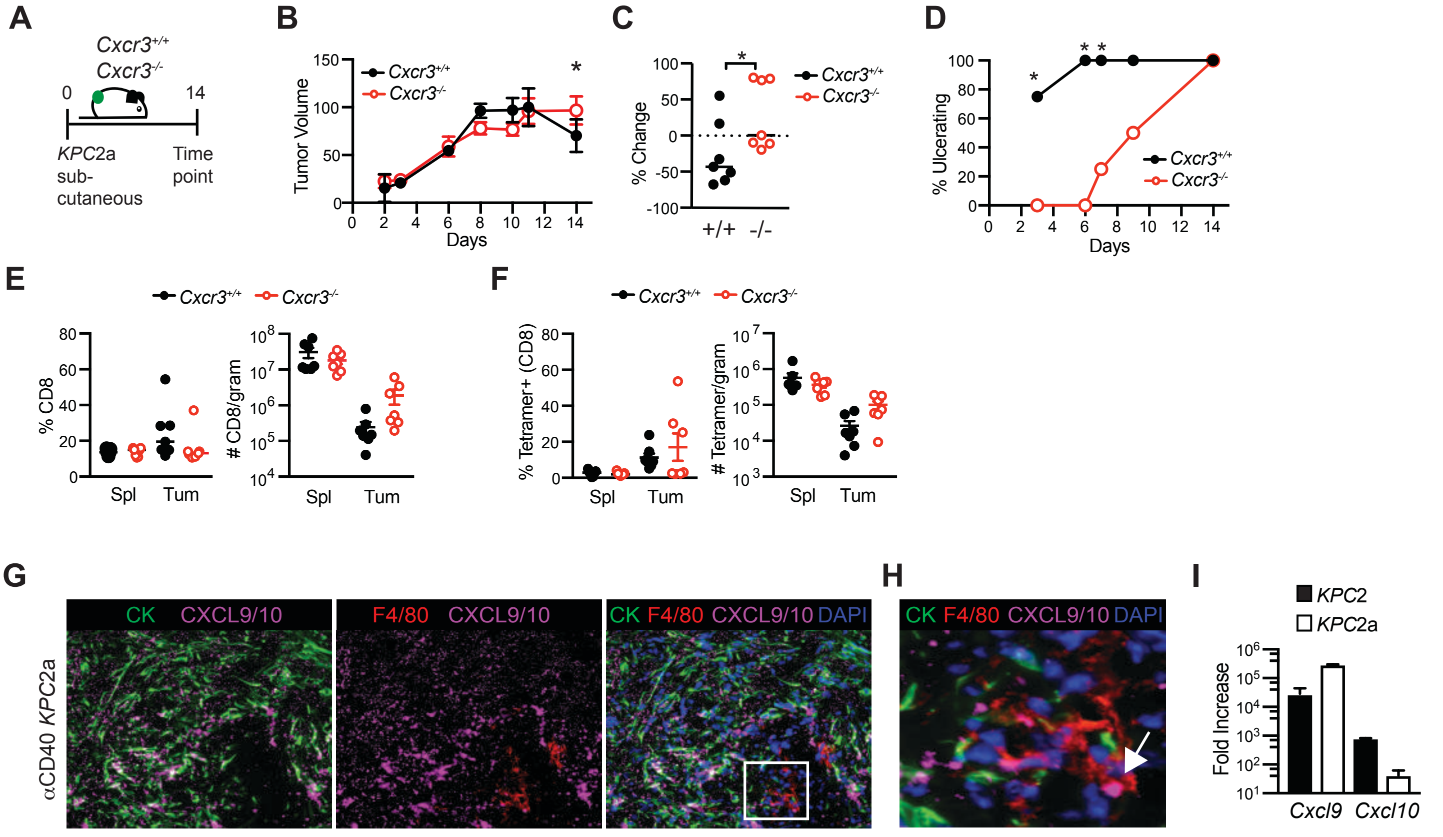

Supplementary Figure 2. T cell migration and accumulation in PDA is Cxcr3-independent.
A) Experimental approach. **B**) Mean *KPC*2a tumor volume following s.c. implantation. n=6 mice per group, pooled from 2 independent experiments. Data are mean ± S.E.M. **p*<0.05, student's T test. **C**) Percent change in tumor volume between 10 and 14 days posttumor from mice in B. **p*<0.05, student's T test. **D**) Percentage of animals with ulcerating tumors from B. **p*<0.05, student's T test. **E**) Frequency of CD8+ T cells gated on CD45+ cells (left) and number of CD8+ T cells per gram tissue (right) at 14 days post s.c. tumor. **F**) Frequency of tetramer+ T cells gated on CD8+ T cells (left) and number of tetramer+ T cells per gram tissue (right) 14 days post s.c. *KPC2a* implantation. Data are mean ± S.E.M. **G**) IF of *KPC2a* (CB+) tumors from mice treated with CD40 agonist (100 μg/mouse) on day 7 posttumor and analyzed on day 14 posttumor. or *KPC2a* (CB+) tumors from mice 7 days post first dose of αPD-L1. **H**) Fold increase in *Cxcl9* and *Cxcl10* by *KPC2* and *KPC*2a cell lines 24 hours post *in vitro* culture with recombinant murine IFNγ was determined by qPCR.

Supplementary Figure 3

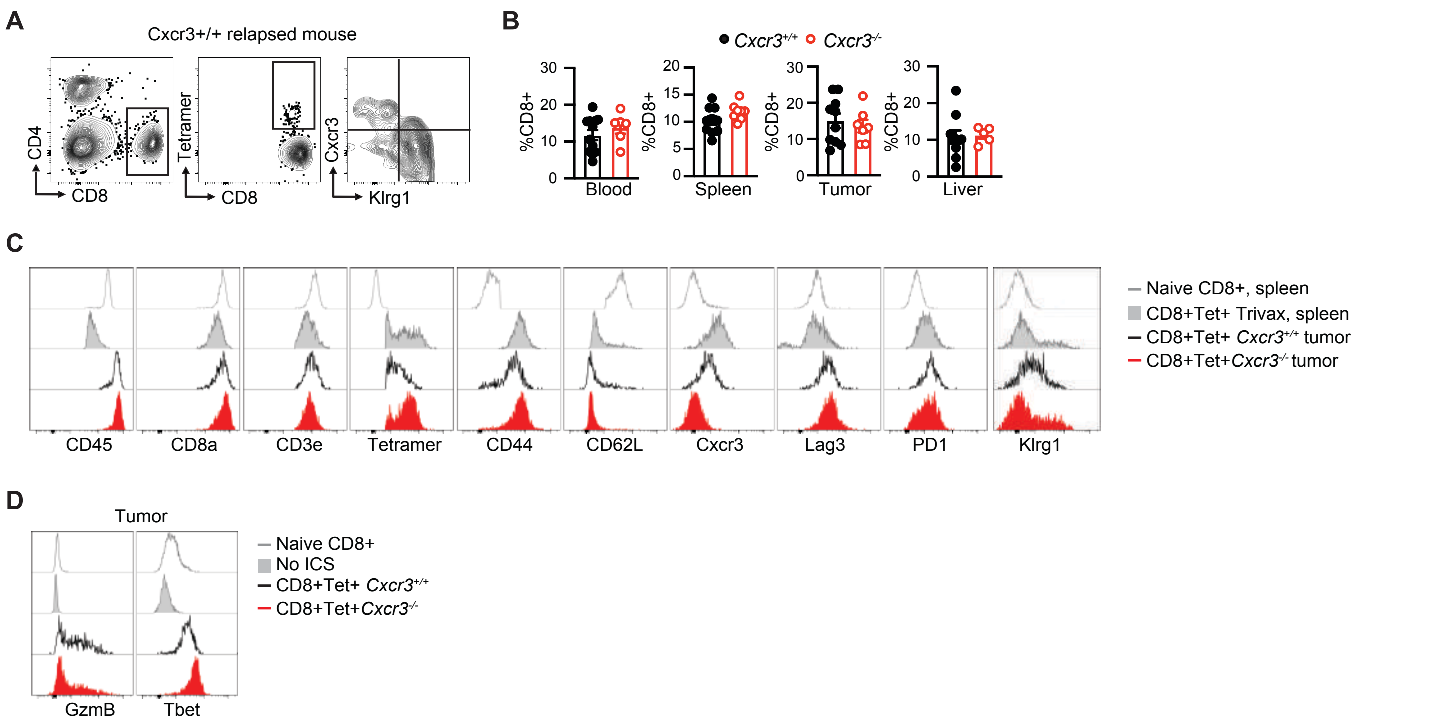

Supplementary Figure 3. Cxcr3 is required for immunotherapy durability and maintenance of peripheral Gzmb+ antitumor T cells. A) Representative gating strategy of persisting tumor-specific T cells on day 35-90 post immunotherapy with αPD-L1 + CD40 agonist. Example is from a relapsed wild type mouse. **B**) Frequency of CD8+ T cells in the indicated tissues from relapsed mice. Data are mean ± S.E.M. **C**) Histograms gated on intratumoral CD8+tetramer+ T cells from relapsed *Cxcr3*$^{+/+}$ vs. *Cxcr3*$^{-/-}$ mice. Naïve splenic CD8+CD44-CD62L+ T cells and splenic CD8+tetramer+ T cells from CD40 agonist + Poly:IC + CB101-109 peptide treated mice on day 7 post vaccination were also included as controls. **D**) Representative histograms of intracellular staining of GzmB and Tbet from *Cxcr3*$^{+/+}$ vs. *Cxcr3*$^{-/-}$ relapsed mice. Naïve splenic CD8+CD44-CD62L+ T cells and a no intracellular stain (ICS) were included as controls. Percentage of animals with ulcerating tumors from B. *$p<0.05$, student's T test.

Supplementary Figure 4

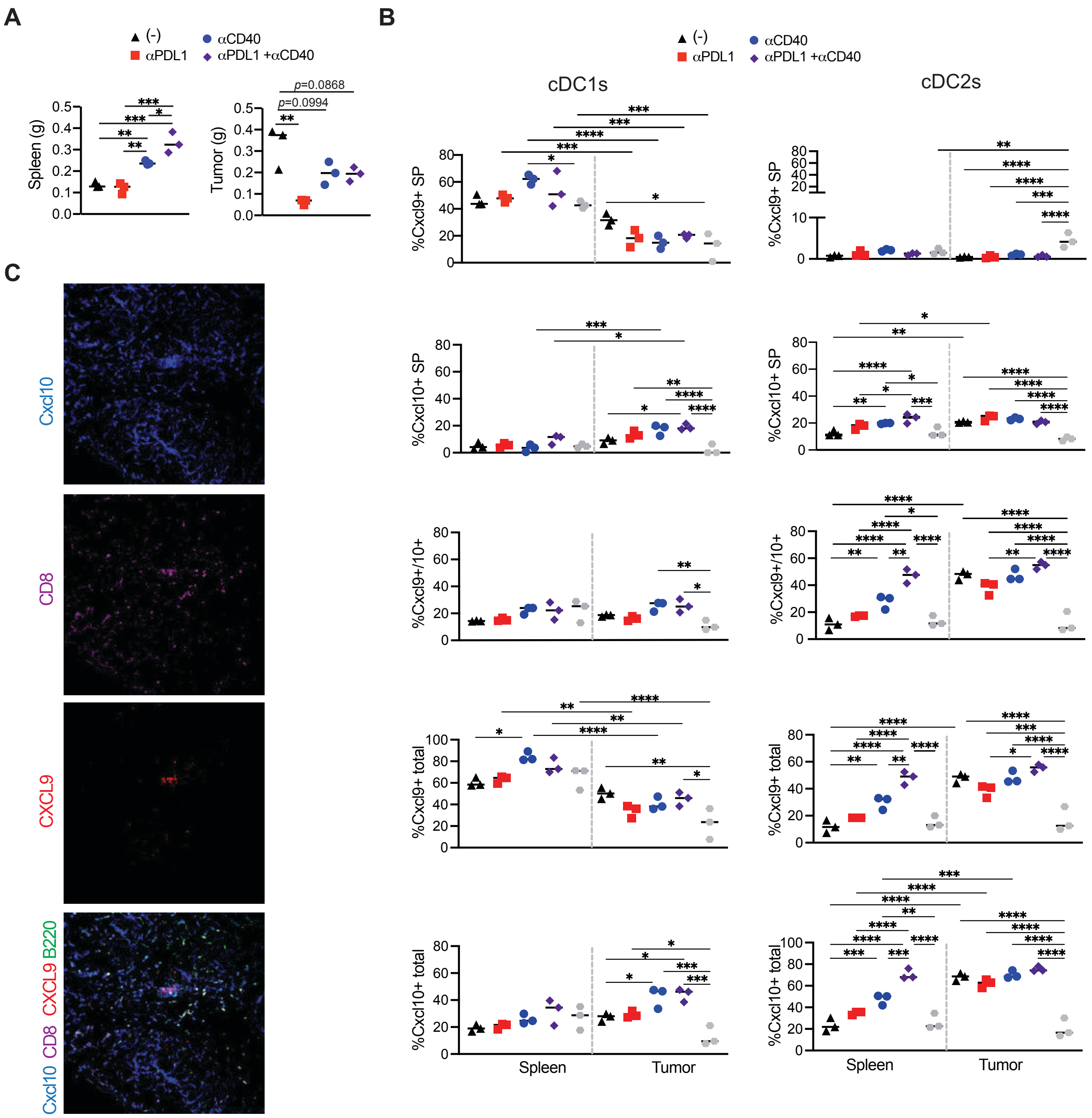

Supplementary Figure 4, continued

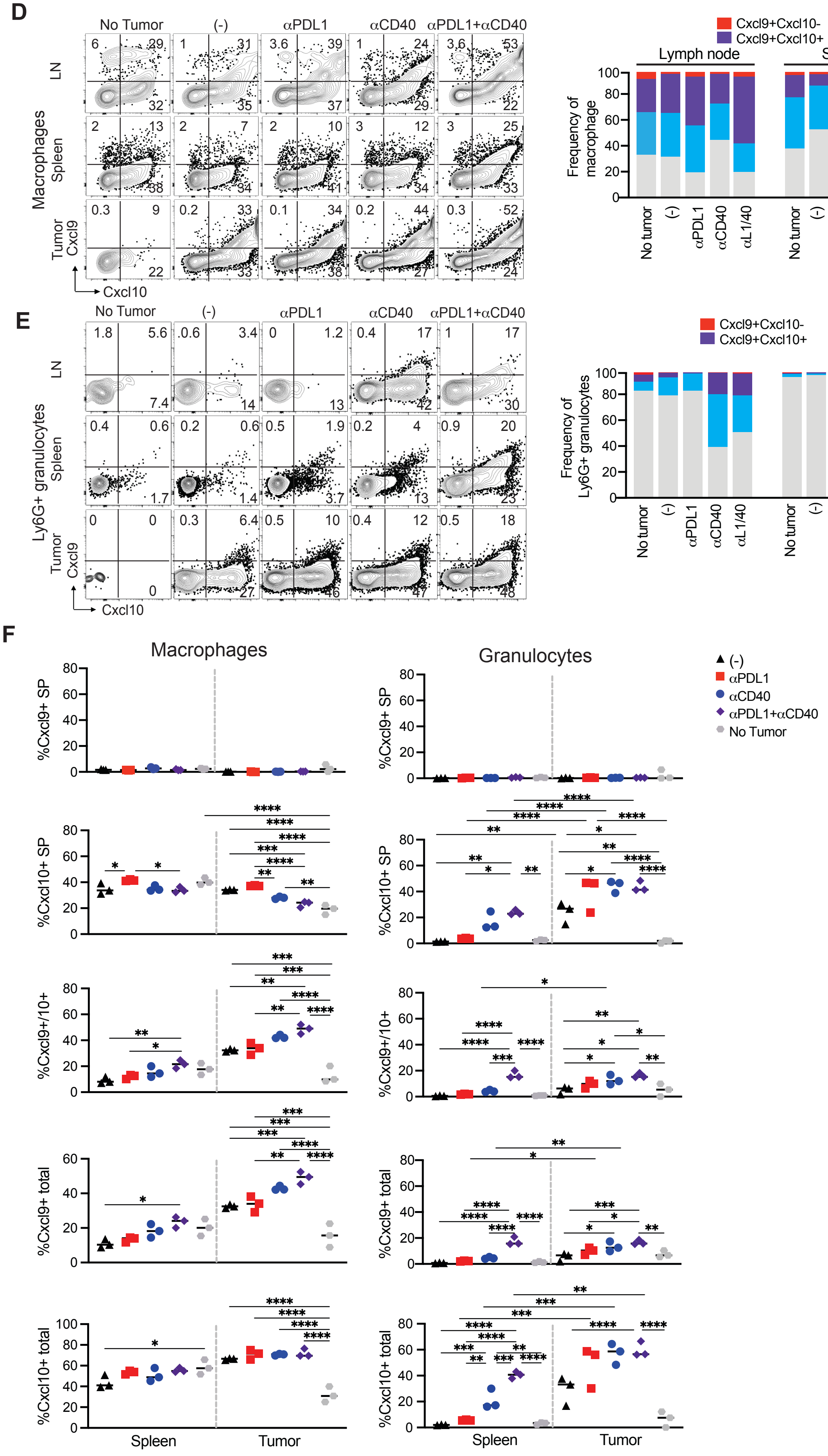

Supplementary Figure 4. Induction of Cxcl9 and Cxcl10 in myeloid cells following immunotherapy. A) Tumor and spleen weight in grams (g) from REX3 mice with orthotopic *KPC2a* PDA on day 14 post tumor implantation, which is day 7 post immunotherapy. **B**) Frequency of cDC1s and cDC2s that are positive for Cxcl9, Cxcl10 or the combination. SP, single positive. ANOVA with a Tukey's posttest. *$p<0.05$, **$p<0.005$, ***$p<0.0001$. **C**) IF for the indicated markers from a tumor from a *KPC*2a tumor-bearing mice. Mean frequency of CD45+ cells that express Cxcl9, Cxcl10 or the combination. **D**) Representative flow plots and graphed data gated on macrophages. **E**) Representative flow plots and graphed data gated on granulocytes. **F**) Frequency of macrophages and Ly6G+ granulocytes positive for Cxcl9, Cxcl10 or the combination. SP, single positive. ANOVA with a Tukey's posttest. *, $p<0.05$, **, $p<0.005$, ***, $p<0.0001$.

Supplementary Figure 5

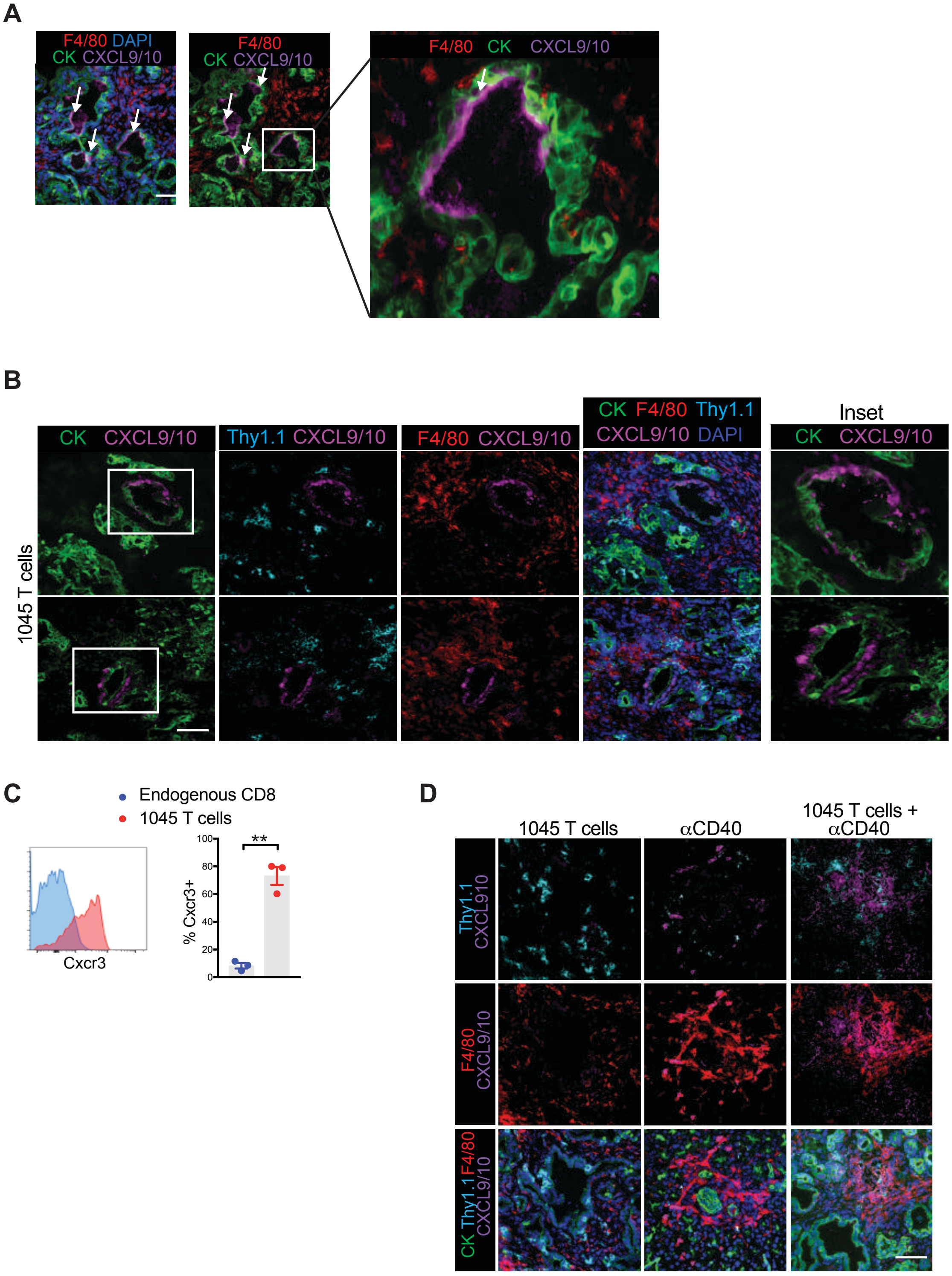

Supplementary Figure 5. Cxcr3 ligands are induced in autochthonous PDA following engineered T cell therapy + CD40 agonist. A) Apical Cxcl9/10 by cytokeratin+ (CK) ductal cells from *KPC* tumors from untreated mice. Scale bar, 50 μm. **B**) Cxcl9/Cxcl10 in *KPC* tumors on day 8 post Thy1.1+ 1045 T cells. Scale bar, 50 μm. **C**) Cxcr3 expression by 1045 T cells prior to transfer and endogenous CD8 T cells from tumors. **, p<0.005. Student's t test. **D**) Co-localization of Cxcl9/Cxcl10 by macrophages in *KPC* tumors on day 8 post 1045 T cells, CD40 agonist, or the combination. Scale bar, 50 μm.
